# Citrullination mediated by PPAD constrains biofilm formation in *P. gingivalis* strain 381

**DOI:** 10.1038/s41522-019-0081-x

**Published:** 2019-02-07

**Authors:** Danielle M. Vermilyea, Gregory K. Ottenberg, Mary E. Davey

**Affiliations:** Department of Oral Biology, College of Dentistry, University of Florida, Gainesville, FL USA

**Keywords:** Biofilms, Microbial genetics

## Abstract

*Porphyromonas gingivalis* is the only known human-associated prokaryote that produces a peptidylarginine deiminase (PPAD), a protein-modifying enzyme that is secreted along with a number of virulence factors via a type IX secretion system (T9SS). While the function of PPAD in *P. gingivalis* physiology is not clear, human peptidylarginine deiminases are known to convert positively charged arginine residues within proteins to neutral citrulline and, thereby, impact protein conformation and function. Here, we report that the lack of citrullination in a PPAD deletion mutant (Δ8820) enhances biofilm formation. More Δ8820 cells attached to the surface than the parent strain during the early stages of biofilm development and, ultimately, mature Δ8820 biofilms were comprised of significantly more cell–cell aggregates and extracellular matrix. Imaging by electron microscopy discovered that Δ8820 biofilm cells secrete copious amounts of protein aggregates. Furthermore, gingipain-derived adhesin proteins, which are also secreted by the T9SS were predicted by mass spectrometry to be citrullinated and citrullination of these targets by wild-type strain 381 in vitro was confirmed. Lastly, Δ8820 biofilms contained more gingipain-derived adhesin proteins and more gingipain activity than 381 biofilms. Overall, our findings support the model that citrullination of T9SS cargo proteins known to play a key role in colonization, such as gingipain-derived adhesin proteins, is an underlying mechanism that modulates *P. gingivalis* biofilm development.

## Biofilms: Porphyromonas gingivalis biofilms limited by citrullination

The oral bacteria *Porphyromonas gingivalis*, implicated in periodontal disease, secretes the citrullination enzyme peptidylarginine deiminase (PPAD), whose surprising role in modulating biofilm formation has been uncovered. Mary Davey and colleagues at the University of Florida show that a strain of *P. gingivalis* with mutated PPAD (which is unable to promote citrullination, or the conversion of arginine residues to citrulline in target proteins) shows enhanced biofilm formation, with mature biofilms from the mutated strain secreting large amounts of proteins and having more cells and deposited extracellular matrix than biofilms from the wild-type strain. Importantly, secreted adhesion proteins known to be involved in *P. gingivalis* colonization were found to be citrullination targets and to be secreted in larger amounts by the PPAD mutant strain. These findings uncover citrullination of *P*. *gingivalis* colonizing factors by PPAD as a key event in biofilm development.

## Introduction

*Porphyromonas gingivalis*, an oral bacterium primarily known for its etiological role in periodontal disease, is implicated in rheumatoid arthritis, which is a common comorbidity in periodontal disease.^1^ Research has shown that generation of anti-citrullinated protein antibodies (ACPAs) is a key feature of rheumatoid arthritis.^2,3^ Interestingly, *P. gingivalis* secretes a peptidylarginine deiminase (PPAD), an enzyme that converts positively charged arginine residues to neutral citrulline residues within peptides and proteins. While humans express five different isotypes (PAD1–4 and PAD 6) that play roles in both health and disease, PPAD is the only known prokaryotic PPAD.^4–12^ Currently, data support the model that citrullination of peptides or proteins by PPAD in the periodontium can lead to a breakdown in tolerance and, thereby, the production of ACPAs, and the development or progression of rheumatoid arthritis.^2,3,5,13–17^ Although the link to rheumatoid arthritis has driven an extensive amount of PPAD research, the role of PPAD in the basic physiology of *P. gingivalis*, in particular its impact during biofilm growth, is still unclear.

*P. gingivalis* is a metabolically atypical anaerobe that utilizes protein substrates as a primary source for energy production and growth. This requires the release of a complex array of proteolytic enzymes into its environment either through direct secretion or indirectly via the release of outer membrane vesicles (OMVs). Secretion of a number of key enzymes, including the proteases known as gingipains (RgpA, RgpB, and Kgp) and PPAD, is accomplished via a Type IX secretion system (T9SS).^18^ Although the role of PPAD in the context of periodontal disease and bacterial physiology is not clear, one model proposes that ammonia, produced as a byproduct of PPAD activity, helps *P. gingivalis* resist acidic cleansing in the mouth.^4,6,19–24^ This working hypothesis is strongly supported by the fact that growth of *P. gingivalis* on protein substrates is inhibited at low pH and citrullination in combination with amino acid fermentation, in particular deamination of lysine and arginine, could generate a highly favorable environment for survival.^25^ Additional research has shown that deleting the gene that encodes PPAD in encapsulated strain W50 inhibits periodontal bone loss in a BALB/c mouse model, while deletion in the non-encapsulated, fimbriated strain ATCC 33277 impairs attachment to and invasion of primary human gingival fibroblasts.^14,26^ Taken together, previous findings indicate that PPAD activity impacts growth, as well as colonization, attachment, and/or invasion of host cells and tissues.

Given the fundamental importance of sessile growth (biofilm formation) to the survival and pathogenic potential of *P. gingivalis*, the goal of this study was to evaluate the effect of PPAD activity during biofilm growth and development. Reports show that *P. gingivalis* can citrullinate a variety of endogenous proteins known to play a role in biofilm formation including a subunit of the major fimbriae (FimA), a subunit of the minor fimbriae (Mfa1), and gingipains (RgpA and Kgp), but the effect of citrullinating these proteins on biofilm development is unclear.^7^ Furthermore, citrullination of free l-arginine in *P. gingivalis* culture by other bacterial arginine deiminases results in the downregulation of fimbriae and subsequent biofilm formation.^27–29^ Although the biofilm-forming strain of *P. gingivalis* used in this study does not have an arginine deiminase, PPAD can citrullinate both peptidylarginine and, to a lesser extent, free L-arginine.^20,23^ Therefore, we hypothesized that PPAD modulates biofilm growth and development by citrullination of peptidylarginine within proteins and/or by regulating the availability of free l-arginine, either directly or indirectly.

To accomplish our goals, we deleted the gene that encodes PPAD in strain 381 (a highly fimbriated and robust biofilm-forming strain that is closely related to strain ATCC 33277, but hyperfimbriated) and investigated the effect of a lack of citrullination on biofilm development. Our analysis discovered that the mutant strain has an enhanced biofilm phenotype resulting in two-fold more biomass. To begin to elucidate the underlying mechanism(s) for this phenotype, we employed different growth conditions along with western analysis, fluorescence microscopy, and electron microscopy (EM). Our studies revealed that the PPAD mutant (Δ8820) secretes copious amounts of protein aggregates and it generates a distinct biofilm architecture; unlike the parent strain, Δ8820 biofilm cells are encased in an extensive lattice-like structure of extracellular matrix. Overall, our findings support the model that citrullination of secreted T9SS cargo proteins, in particular gingipain-derived adhesins, impacts the release and localization of these proteins, which in turn alters cell–cell interactions and biofilm matrix development.

## Results

### Deletion of PGF_00008820, the gene encoding PPAD in strain 381, enhances biofilm formation

**Fig. 1:**
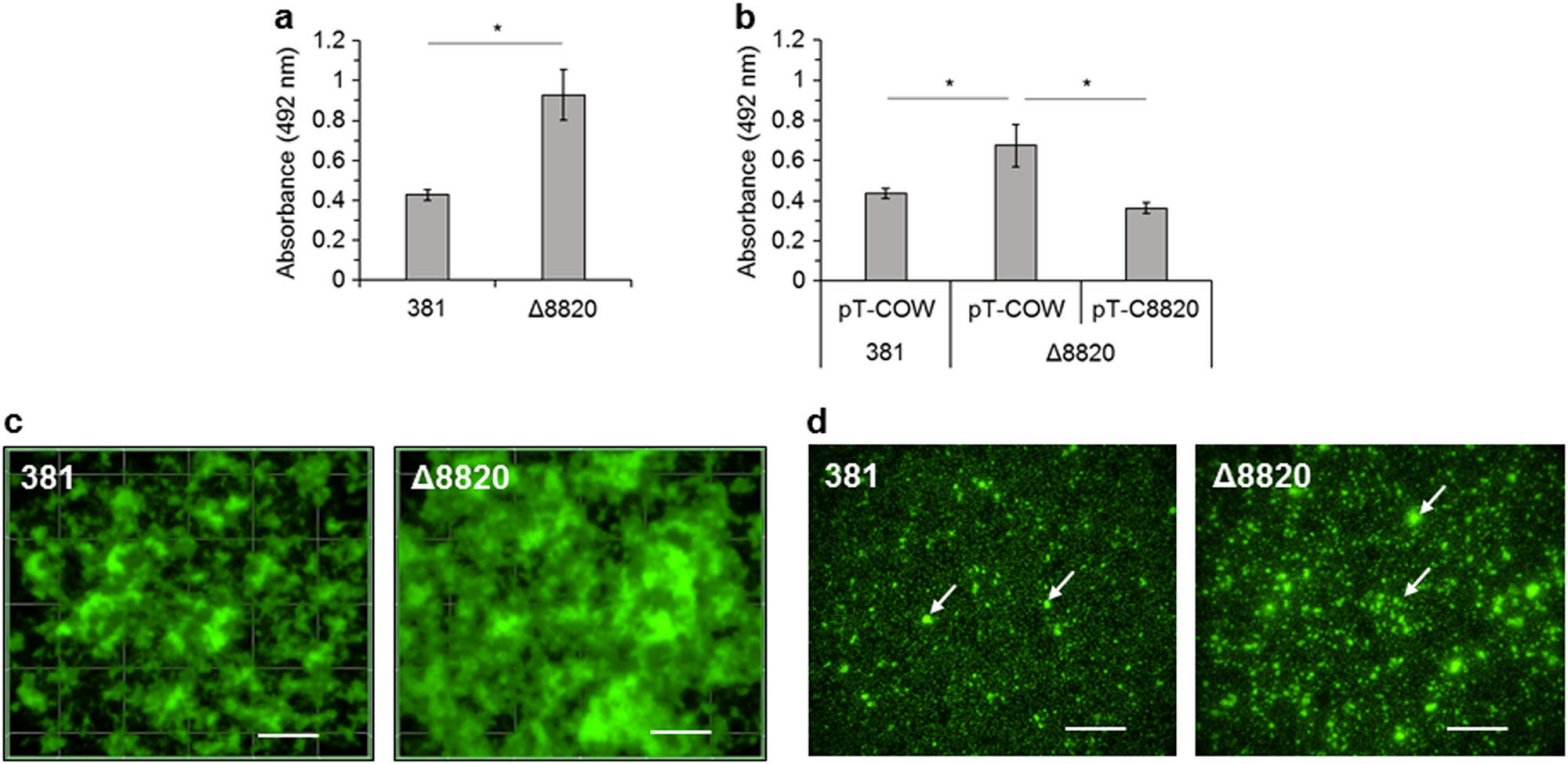
Deletion of the gene encoding PPAD enhances biofilm formation. **a** 381 and Δ8820 biomass after 24 h was quantified by staining with safranin. **b** 24 h biomass of empty vector controls (381 pT-COW and Δ8820 pT-COW) and complemented Δ8820 (Δ8820 pT-C8820) was quantified by staining with safranin. Data in **a** and **b** are averages of three independent experiments (*n* = 9). Error bars represent the standard deviation. The data in **a** were analyzed using the Student’s two-tailed *t*-test. The data in **b** were analyzed by ANOVA with Bonferroni post tests. *p < 0.05. **c** Top-down view of 24 h biofilms stained with SYTO 9 and propidium iodide. **d** Attached cells 2 h after setting up a biofilm assay stained with SYTO 9 and propidium iodide. Arrows indicate examples of cell–cell aggregates. Scale bar: 50 µm

Previous findings show that removal of free l-arginine from the environment by arginine deiminases inhibits biofilm formation of *P. gingivalis*, while addition of l-arginine can enhance attachment.^27–29^ Although these findings are specific to free l-arginine, they demonstrate the importance of l-arginine in and the impact of arginine deiminases on *P. gingivalis* biofilm formation. Therefore, we hypothesized that deletion of the gene encoding PPAD, a PPAD, in hyperfimbriated strain 381 would affect peptidylarginine and/or l-arginine availability and, thereby, biofilm formation. To test this hypothesis, we first generated a PPAD deletion mutant in strain 381 (Δ8820), confirmed the depletion of enzymatic activity by colorimetric assay, and determined that there was no change in growth rate (Supplementary Figures 1 and 3). We measured biofilm formation by safranin staining and found that the PPAD deletion mutant had an enhanced biofilm phenotype with an average absorbance at 492 nm (*A*_492_) of 0.93 ± 0.13 compared to an *A*_492_ of 0.43 ± 0.03 for the wild type (Fig. 1a). We then complemented strain Δ8820 *in trans* (Δ8820 pT-C8820) and transformed strains 381 and Δ8820 with the empty vector, pT-COW, as controls. Complementation of Δ8820 restored PPAD enzymatic activity (Supplementary Figure 2). Although complementation resulted in a greater rate of enzymatic activity in Δ8820 pT-C8820, the concentration of citrulline measured in the stationary cultures was the same in 381 pT-COW and Δ8820 pT-C8820 (Supplementary Figures 2 and 3). When we measured biofilm formation, we found that the presence of the pT-COW control plasmid lowered the *A*_492_ of Δ8820 to 0.68 ± 0.10 from 0.93 ± 0.13, but Δ8820 pT-COW biomass was still significantly greater than that of 381 pT-COW (Fig. 1b). Importantly, when the deletion mutant was complemented, biofilm formation was restored to wild-type levels (Fig. 1b). Overall, these findings show that deletion of the gene encoding PPAD in strain 381 enhances biofilm formation, while expression of PPAD inhibits biofilm development.

**Fig. 2:**
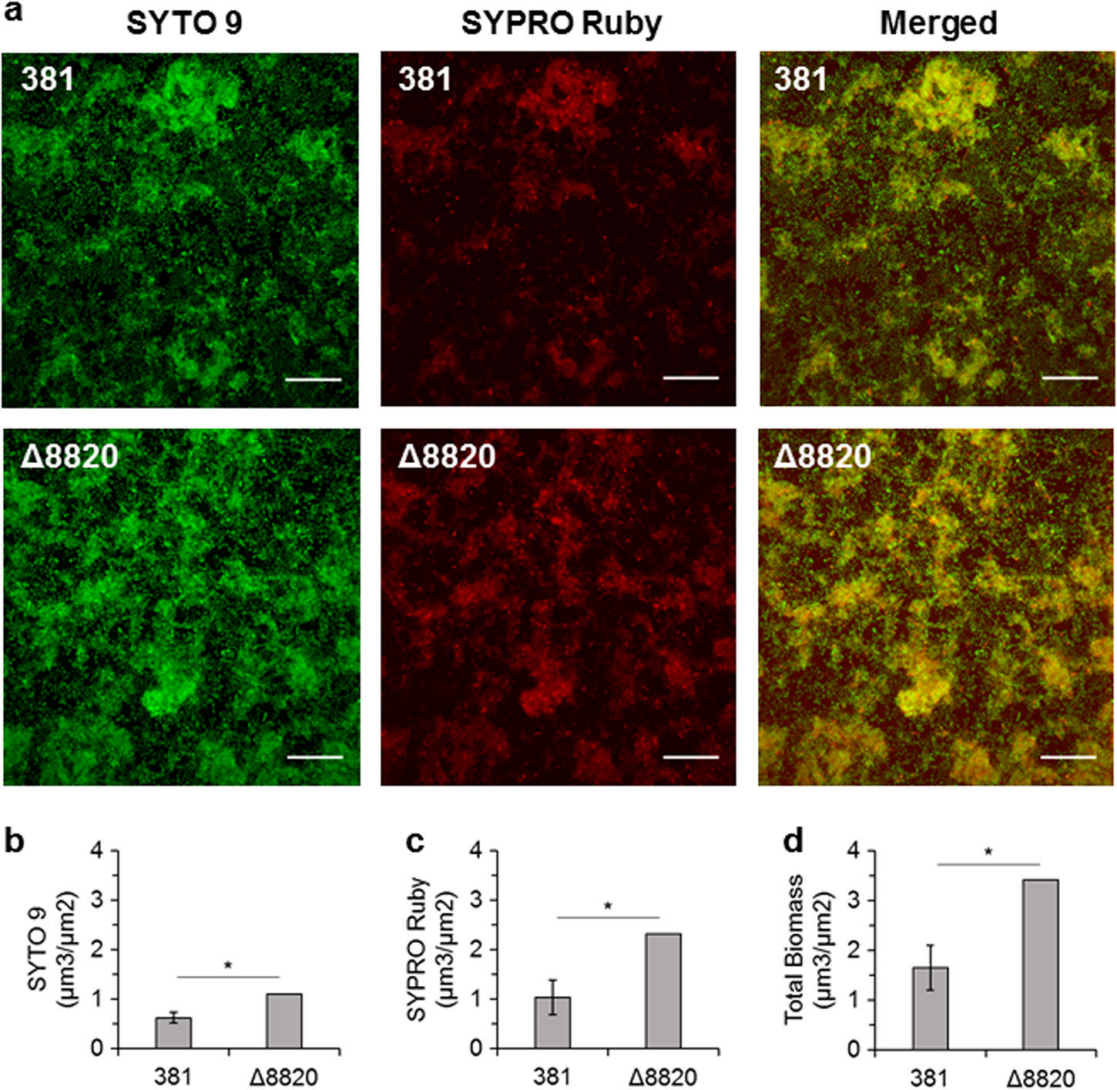
Δ8820 biofilms are comprised of more bacterial cells and protein than 381 biofilms. **a** 381 and Δ8820 were grown for 24 h on glass, stained with SYTO 9 (cells) and SYPRO Ruby (extracellular protein), and images were acquired by CLSM. Images are a top-down view of stacked maximum intensity projection images. Scale bar: 20 µm. **b** SYTO 9, **c** SYPRO Ruby, and **d** total biomass were quantified using Comstat2. Data are averages of two replicates (*n* = 2). Error bars represent the standard deviation. The data were analyzed using the Student’s two-tailed *t*-test. *p < 0.05

Biofilm architecture was characterized by staining with SYTO 9 to stain all bacterial cells and propidium iodide to stain only dead cells. Fluorescence microscopy confirmed that Δ8820 biofilms were comprised of more biomass than 381 biofilms (Fig. 1c, 2d). Additionally, Δ8820 biofilms had larger microcolonies with less void space between them (Fig. 1c). We also examined surface attachment by cells during the early stages of biofilm development. Two hours after setting up the static biofilm assay, wells were washed and attached cells were examined by staining with SYTO 9 and propidium iodide. Compared to cell attachment by strain 381, more Δ8820 cells attached to the well subsurface as punctate aggregates (Fig. 1d). No differences in viability were observed between 381 and Δ8820. Biofilm composition was also evaluated by staining with SYTO 9 and SYPRO Ruby (extracellular protein detection). The biomass of Δ8820 biofilms was comprised of more cells and more protein (Fig. 2b–d). The ratio of extracellular protein to cells (SYPRO Ruby to SYTO 9) was 1.62 ± 0.28 for 381 and 2.11 ± 0 for Δ8820, indicating that Δ8820 biofilms contained more extracellular protein per cell, thereby, suggesting a defect in the release of proteins into the surroundings. Taken together, the data indicate that the enhanced biofilm phenotype of the PPAD deletion mutant is due to greater cell–cell interactions and attachment in conjunction with greater accumulation of cell surface and biofilm matrix protein.

### Deletion of the gene encoding PPAD results in increased matrix production

**Fig. 3:**
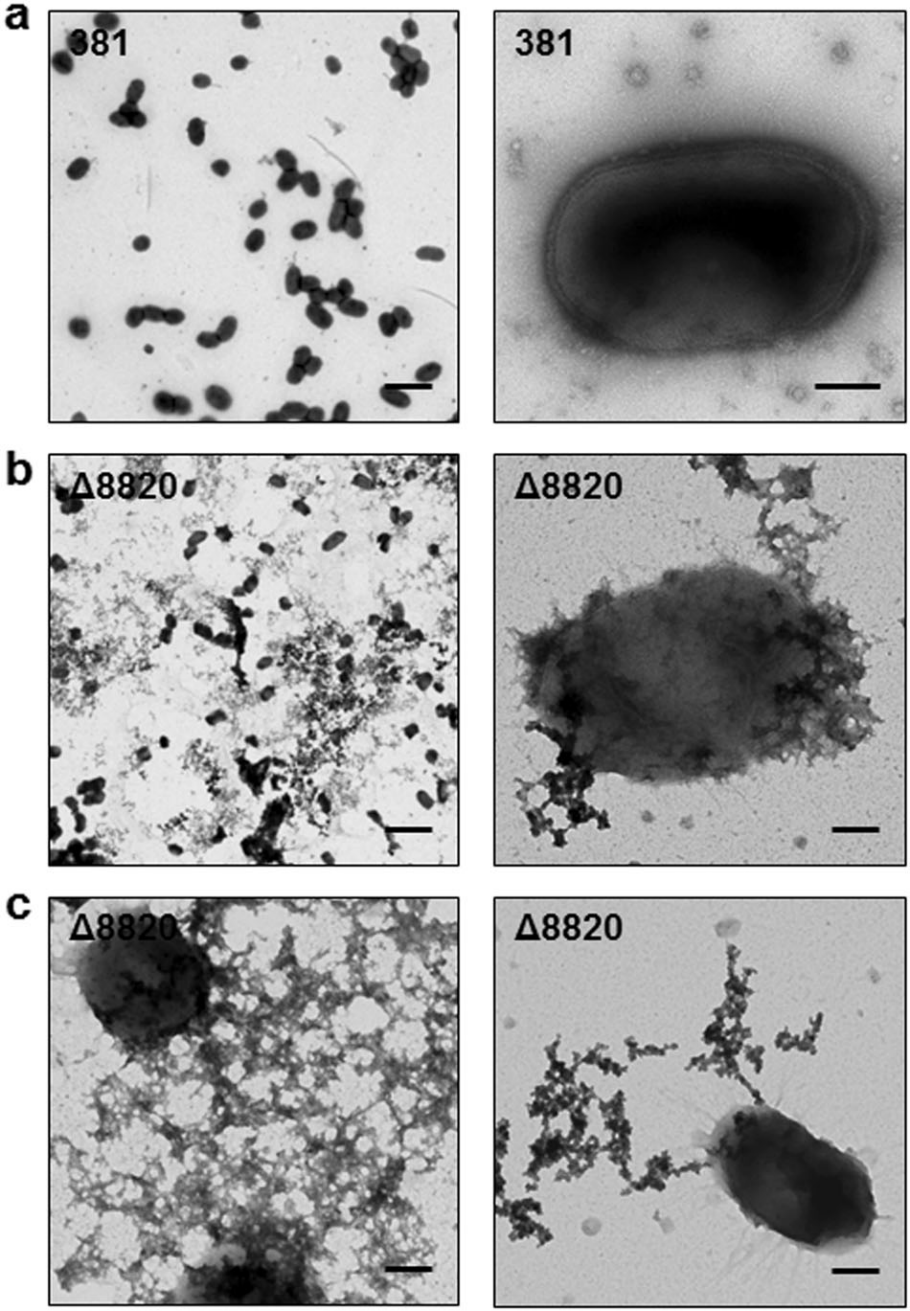
Δ8820 cells produce a surface-associated extracellular substance that is not present on 381 cells. Bacterial cells from **a** 381 and **b**, **c** Δ8820 colony biofilms grown on plates were negatively stained with 0.5% aqueous uranyl acetate and then imaged by TEM. **a** 381 displayed typical cell morphology. Scale bar: (left) 2 µm and (right) 200 nm. **b** Δ8820 cells were (left) surrounded by and (right) coated with an extracellular substance that could be stained by uranyl acetate. Scale bar: (left) 2 µm and (right) 200 nm. **c** The extracellular substance in Δ8820 samples was capable of (left) forming a meshwork around cells and (right) appeared to extrude from specific sites at the cell surface. Scale bar: 200 nm

**Fig. 4:**
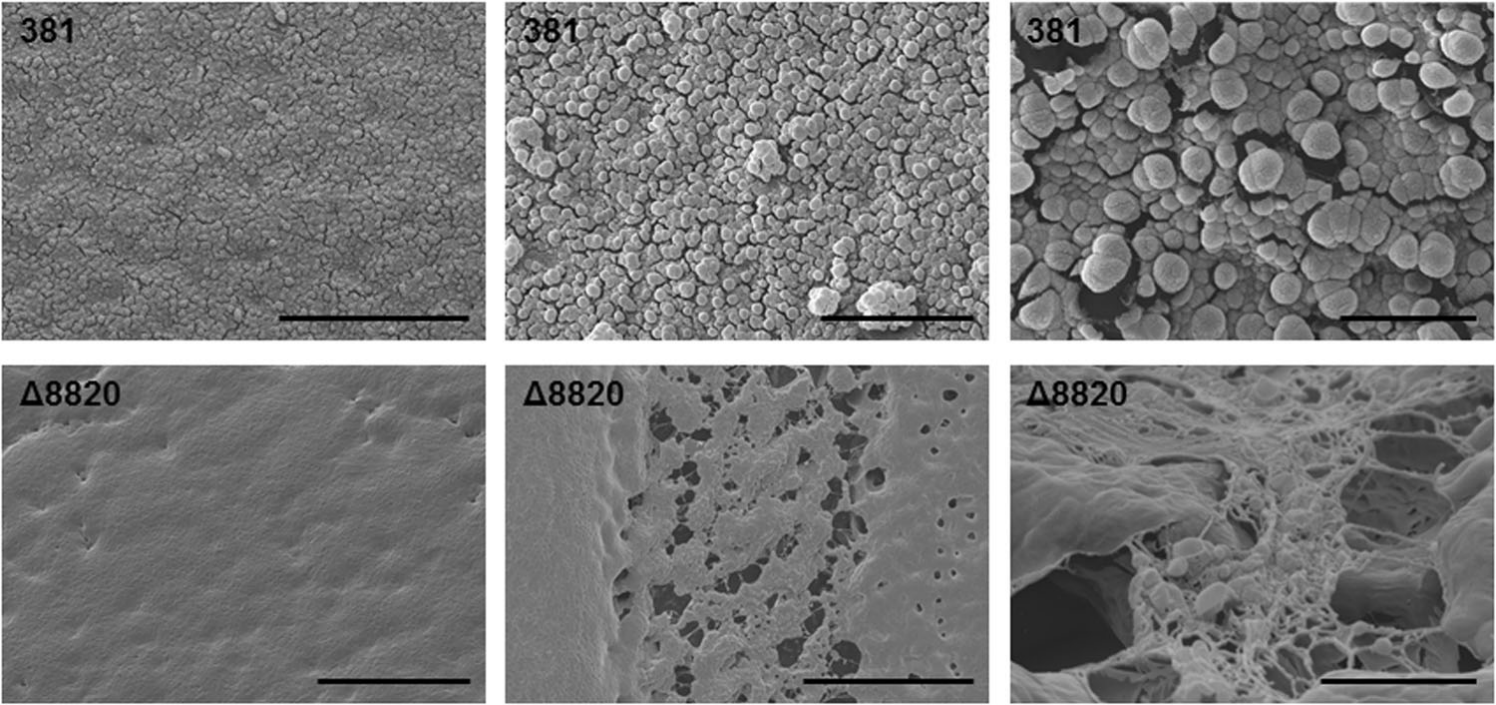
Deletion of the gene encoding PPAD results in increased matrix production. 381 and Δ8820 colony biofilms grown on plates were imaged by Cryo-SEM. Bacterial cells were visible on the surface and within 381 colony biofilms. The surface of Δ8820 colony biofilms were smooth and cells within the biofilms were coated or encased in a matrix-like substance. Scale bar: (top left) 10 µm, (top middle) 5 µm, (top right) 1 µm, (bottom left) 20 µm, (bottom middle) 20 µm, (bottom right) 2 µm

Localization of PPAD and PPAD enzymatic activity has been examined in several strains of *P. gingivalis*.^4,7,8^ To examine the localization of PPAD enzymatic activity in strain 381, we measured enzymatic activity associated with the cell surface and the supernatant using a colorimetric assay. The majority of enzymatic activity, 69 ± 5.4%, in 381 was cell-surface associated, while 31 ± 5.4% was associated with the supernatant (Supplementary Figure 4a). These results were in line with previous findings for other strains of *P. gingivalis*.^4^ Although the majority of enzymatic activity was cell associated, almost all of the citrulline that was detectable using the colorimetric assay was found in the supernatant after 24–36 h (Supplementary Figure 4b). Therefore, our findings implicated secreted or extracellular proteins as substrates of PPAD in monoculture. Next, we performed transmission electron microscopy (TEM) of negatively stained cells to investigate and characterize the surface structures and extracellular environment of 381 and Δ8820 cells from colony biofilms grown on blood agar plates. TEM revealed that Δ8820 samples produced an abundance of negatively stained extracellular substance that was not present in 381 samples (Fig. 3). Upon closer observation, the surface of mutant cells was decorated by this substance, which extended from the cell surface as fibers (Fig. 3b, c). Further, the fibers were capable of forming a meshwork around mutant cells and appeared to extrude from discrete cell surface sites (Fig. 3c). Since uranyl acetate primarily binds lipids and proteins, and PPAD activity is tightly linked to secretion of T9SS cargo proteins, our working hypothesis was that the extracellular substance produced by Δ8820 was composed of protein and that the enhanced biofilm phenotype of Δ8820 was due, in part, to greater matrix production. To investigate this hypothesis, we performed cryo scanning electron microscopy (Cryo-SEM) on colony biofilms grown on blood agar plates in order to retain the conformation of the biofilms. 381 colony biofilms contained easily identifiable bacterial cells (Fig. 4, top). In contrast, Δ8820 colony biofilms had a smooth, dimpled surface and cells within these biofilms were coated with or encased in a matrix-like substance (Fig. 4, bottom). These observations support our hypothesis that deletion of PPAD alters the biofilm matrix.

### The WT can citrullinate recombinant gingipain adhesin domains in vitro

Given the protein-centric physiology of *P. gingivalis* and our above findings, we hypothesized that the altered matrix of Δ8820 biofilms was composed of or due to the accumulation of secreted protein(s). Studies show that a variety of secreted proteins are predicted to be citrullinated including gingipains RgpA and Kgp, fimbriae subunits FimA and Mfa1, and other outer membrane and heme-binding proteins.^7^ Fimbriae are major surface structures that mediate cell–cell interactions and surface attachment in *P. gingivalis*.^30–34^ Studies have shown that removal of arginine from the environment by arginine deiminases inhibits fimbriae production and subsequent biofilm formation by *P. gingivalis*.^27–29^ As a result, we hypothesized that the enhanced biofilm formation by Δ8820 was due to an increase in fimbriae on the cell surface. Surprisingly, western blot analysis of both major (FimA) and minor (Mfa1) fimbriae proteins in 381 and Δ8820 biofilms showed no differences in expression (Supplementary Figure 5b). Similarly, no differences were observed between 381 and Δ8820 planktonic cell cultures (Supplementary Figure 5c). Therefore, the enhanced biofilm phenotype of the PPAD deletion mutant is not due to an increase in fimbriae on the cell surface.

**Fig. 5:**
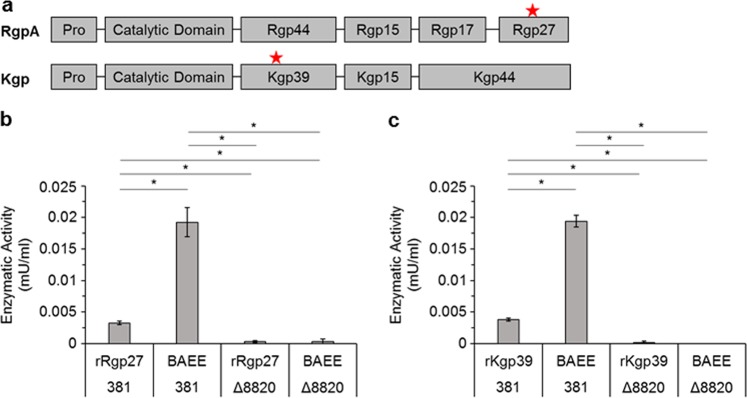
381 citrullinates gingipain-derived adhesin proteins. **a** 381 and Δ8820 cell lysates were analyzed by mass spectrometry to identify citrulline residues in 381. Mass spectrometry predicted a citrulline residue within adhesin domain Rgp27 of gingipain RgpA and within adhesin domain Kgp39 of gingipain Kgp (red stars). **b**, **c** Recombinant Rgp27 and Kgp39 were expressed in *E. coli* and then used as substrates in the PPAD enzymatic activity assay. 381 was able to citrullinate both **b** rRgp27 and **c** rKgp39 in vitro, whereas Δ8820 was unable to citrullinate rRgp27, rKgp39, or BAEE (positive control). Data in **b** and **c** are representative of two independent experiments showing similar results (*n* = 3). Error bars represent the standard deviation of technical replicates. The data were analyzed by ANOVA with Bonferroni post tests. *p < 0.05

In order to identify citrullinated proteins, we made attempts to use an anti-citrulline (modified) detection kit (EMD Millipore #17-347B) as previously described, but this proved unsuccessful.^5,13,16^ Alternatively, we analyzed stationary phase 381 and Δ8820 cell lysates by mass spectrometry to identify citrulline residues. Under the conditions tested, neither FimA nor Mfa1 were predicted to be citrullinated. However, there were two proteins in which a citrulline residue was predicted in all three 381 samples and in none of the Δ8820 samples: RgpA and Kgp (Fig. 5a, Supplementary Figure 6). RgpA and Kgp are gingipains, which are proteases that cleave at arginine residues or lysine residues, respectively. RgpA and Kgp are transcribed as precursor proteins containing a prodomain, the catalytic or proteolytic domain, and the adhesin domain, before ultimately being proteolytically processed and glycosylated.^35,36^ The adhesin domains of RgpA and Kgp are autoprocessed into smaller adhesin proteins Rgp44, Rgp15, Rgp17, Rgp27, Kgp39, Kgp15, and Kgp44.^36^ Interestingly, the predicted citrulline residues were within the adhesin domains, specifically within adhesin proteins Rgp27 and Kgp39 (Fig. 5a). However, using mass spectrometry to identify citrulline residues is difficult. Deamidation and citrullination both produce a 1 Da shift; if a peptide contains both an asparagine or glutamine that can be deamidated and an arginine that can be citrullinated, it is not possible to determine which modification produced the shift.^7^ Therefore, to verify our mass spectrometry findings, we tested the ability of 381 to citrullinate these proteins in vitro. To perform these experiments, recombinant Rgp27 and Kgp39 (rRgp27 and rKgp39) were expressed in *E. coli* (Supplementary Figures 7 and 8) and used as substrates in a colorimetric assay that measures citrullination. Strain 381 was able to citrullinate both rRgp27 and rKgp39 at low levels, whereas, Δ8820 was unable to citrullinate rRgp27, rKgp39, or BAEE (Fig. 5b, c). Overall, although some of the previously identified PPAD targets were not detected in strain 381 under these growth conditions, our data support previous findings that *P. gingivalis* can citrullinate RgpA and Kgp and further shows that *P. gingivalis* can specifically citrullinate gingipain-derived adhesins in vitro.

### PPAD mutant biofilms contain more gingipain-derived adhesin proteins that localize to the matrix

**Fig. 6:**
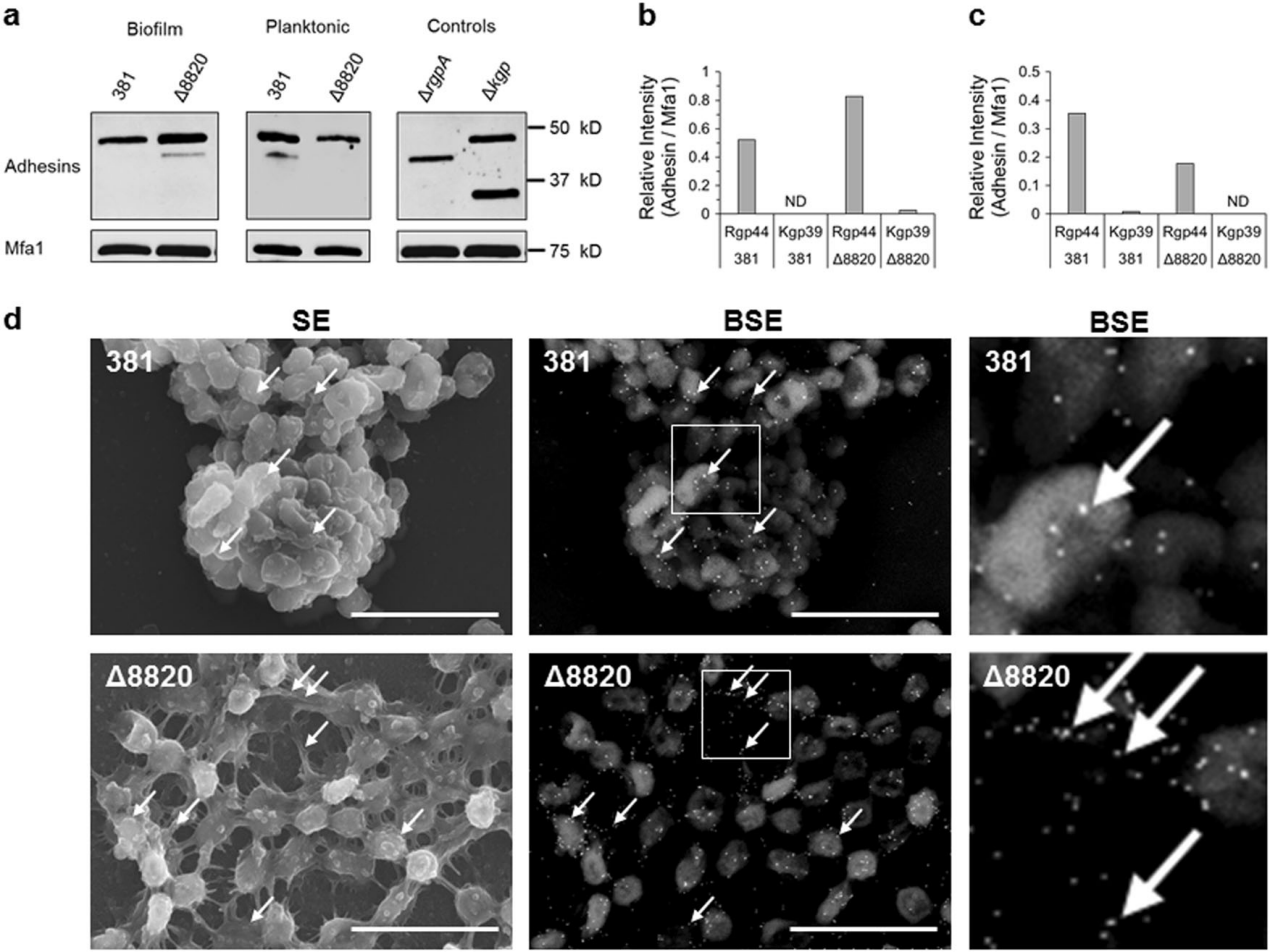
Δ8820 biofilms contain more gingipain-derived adhesin proteins than 381 biofilms. **a** Western blot analysis of biofilm lysates and planktonic cell lysates using an anti-adhesin primary antibody. Δ*rgpA* and Δ*kgp* planktonic cell lysates were used to determine the specific adhesin proteins (from top to bottom: Rgp44, Kgp39, Rgp27). Mfa1 was used as a loading control. All identified bands are shown. Samples derive from the same experiment and the blots were processed in parallel. **b**, **c** Quantification of **b** biofilm and **c** planktonic blots shown in **a**. ND, none detected **d** Suspended 381 and Δ8820 colony biofilms were treated with the anti-adhesin primary antibody and then images were acquired by immunogold labeling and SEM. Gingipain-derived adhesin proteins localized to the surface of 381 cells, the surface of Δ8820 cells, and the matrix around Δ8820 cells. Arrows indicate representative colloidal gold particles. SE secondary electron, BSE ackscatter electron. Scale bar: 2 µm

*P. gingivalis* strain 381 cultures expressing PPAD and, thereby, containing peptidylcitrulline formed less biofilm than Δ8820. Additionally, strain 381 citrullinated the gingipain-derived adhesin proteins, which play a central role in *P. gingivalis* attachment.^37–39^ Therefore, we hypothesized that 381 biofilms contain fewer adhesin proteins than Δ8820 biofilms. To test this, 381 and Δ8820 biofilm lysates were examined by western blot using an anti-adhesin primary antibody (kindly provided by Dr. Mike Curtis, King’s College London, London, UK). This anti-adhesin antibody was generated using the entire recombinant RgpA adhesin domain. The RgpA adhesin domain has high sequence similarity to the adhesin domain of Kgp, allowing the antibody to bind to all of the RgpA and Kgp adhesin proteins.^38,40^ Although a band corresponding to Rgp27 was not observed under the conditions tested, western blot analysis revealed bands corresponding to Rgp44 and Kgp39 in 381 and Δ8820 biofilm lysates (Fig. 6a). Δ8820 biofilm lysates contained more Rgp44 and, to a lesser extent, Kgp39 than 381 biofilm lysates (Fig. 6a, b). The planktonic cell fraction showed the opposite; 381 planktonic cell lysates contained more Rgp44 and Kgp39 than Δ8820 planktonic cell lysates (Fig. 6a, c). Overall, western blot analysis of 381 and Δ8820 showed that Δ8820 biofilms contained more gingipain-derived adhesin proteins suggesting that citrullination of proteins that play a role in cell attachment modulates biofilm formation and development.

Because Δ8820 biofilms contain more gingipain-derived adhesin proteins and more matrix than 381, we hypothesized that the gingipain-derived adhesin proteins are part of the biofilm matrix. To examine where adhesin proteins are localized, cells from 381 and Δ8820 colony biofilms were examined by TEM and scanning electron microscopy (SEM) followed by labeling with the primary anti-adhesin antibody and immunogold labeling. Immunogold labeling and TEM proved difficult due to a lack of contrast between the colloidal-gold-labeled secondary antibodies and the heavily stained extracellular aggregates. However, we could observe that anti-adhesin antibodies localized to the surface of the cells and to OMVs (Supplementary Figure 9). To supplement the TEM, we performed immunogold labeling and SEM, which does not require a negative stain. Under these conditions, we observed that gingipain-derived adhesin proteins primarily localized to the surface of 381 cells, while adhesins localized to Δ8820 cells and to matrix-like structures surrounding Δ8820 cells (Fig. 6d). Furthermore, gingipain-derived adhesin proteins localized to OMVs were more often observed in 381 than in Δ8820 (Supplementary Figure 10). Overall, our data indicate that Δ8820 biofilms have more matrix and the matrix contains more gingipain-derived adhesin proteins. Additionally, preliminary observations of fewer adhesin proteins in the OMV fractions of Δ8820 may suggest that OMVs, or improperly released OMVs containing adhesin proteins, in part, account for the accumulation of adhesin proteins in Δ8820 biofilms, but further work is required to support this working hypothesis.

### Deletion of PPAD decreases secreted Rgp enzymatic activity and increases Rgp and Kgp enzymatic activity within biofilms

**Fig. 7:**
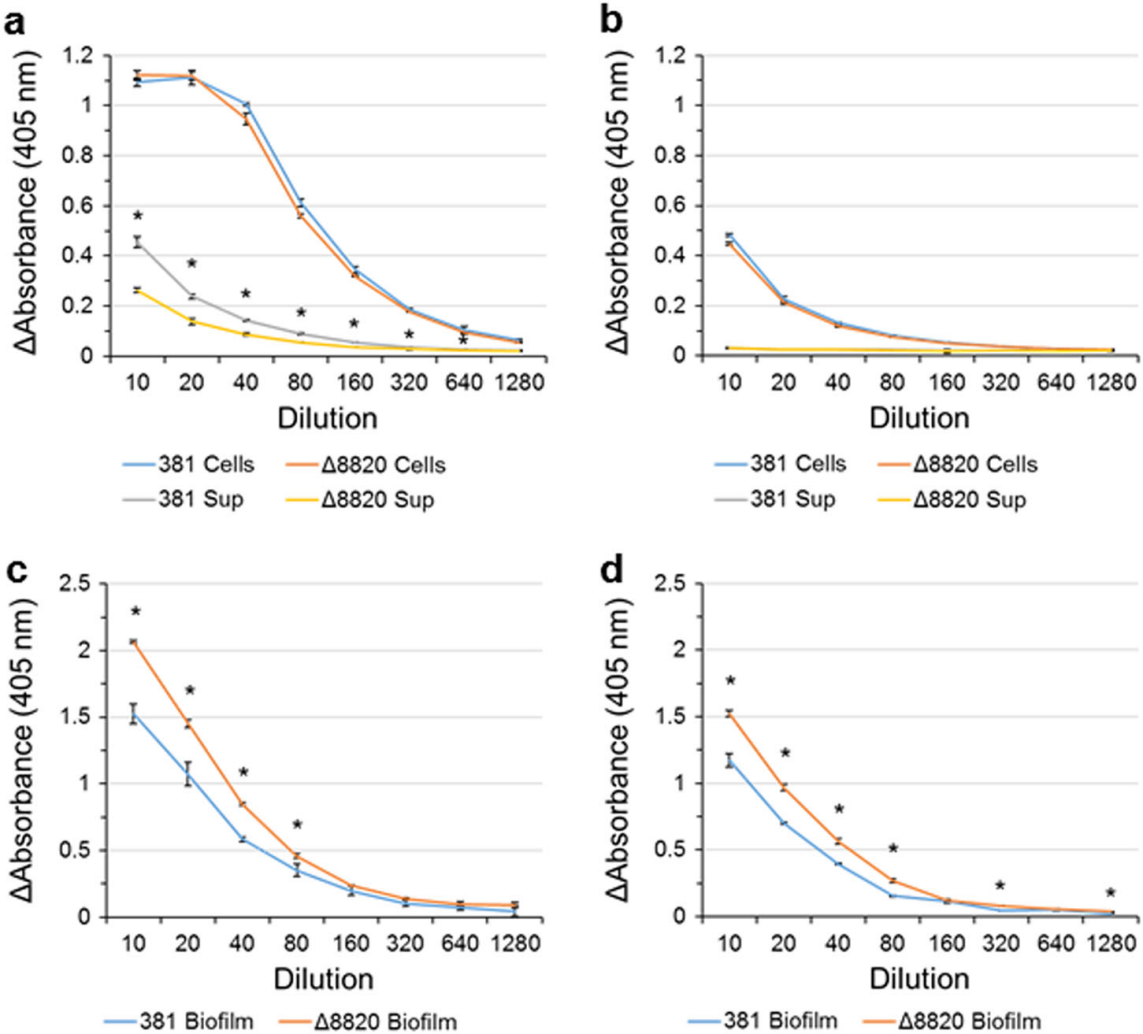
Deletion of PPAD decreases secreted Rgp enzymatic activity and increases Rgp and Kgp enzymatic activity in biofilms. Rgp enzymatic activity in **a** planktonic cultures and **c** biofilm lysates, and Kgp enzymatic activity in **b** planktonic culture and **d** biofilm lysates was measured using a colorimetric assay. **a** Less Rgp activity was measured in the supernatants of Δ8820 cultures. **b** There were no differences in Kgp activity between 381 and Δ8820. Both **c** Rgp and **d** Kgp enzymatic activities were greater in Δ8820 biofilm lysates than 381 biofilm lysates. Data are representative of three independent experiments showing similar results (*n* = 3). Error bars represent the standard deviation of technical replicates. The data were analyzed using the Student’s two-tailed *t*-test. *p < 0.05

Given that the adhesin domains are initially transcribed as part of precursor proteins, whether or not citrullination of the adhesin domains has an effect on gingipain activity was not clear. To test the effect of deleting PPAD on gingipain enzymatic activity, we used a colorimetric assay to measure Rgp and Kgp enzymatic activity of 381 and Δ8820 planktonic cells, culture supernatants, and biofilm lysates. There was no difference in cell-associated Rgp activity between 381 and Δ8820, but the supernatant-associated Rgp activity was lower in Δ8820 than 381 (Fig. 7a). There was no difference in cell-associated or supernatant-associated Kgp activity between 381 and Δ8820 (Fig. 7b). However, there was greater Rgp and Kgp enzymatic activities in Δ8820 biofilm lysates than 381 biofilm lysates (Fig. 7c, d). Therefore, our data show that a lack of PPAD activity decreases the Rgp enzymatic activity in the supernatant while increasing the biofilm-associated Rgp and Kgp activities, indicating that citrullination determines whether these enzymes are retained in the biofilm or released.

## Discussion

*P. gingivalis* is an oral bacterium strongly implicated in the etiology of adult periodontal disease due, in part, to its ability to colonize and persist within the subgingival biofilm.^2,25,41,42^ Identifying and understanding mechanisms that mediate cell attachment and surface attached growth is therefore of fundamental importance to understanding the pathogenic potential of *P. gingivalis*. Although studies have shown that *P. gingivalis* can citrullinate endogenous proteins, the effect of these citrullinated proteins on its physiology remains unclear. Here, we showed that deletion of the gene encoding PPAD prevented the citrullination of proteins, which enhanced biofilm formation in monoculture by enhancing cell–cell interactions, attachment, and matrix production. Surprisingly, this phenotype was not due to an increase in major or minor fimbriae on the cell surface, but instead due, at least in part, to the accumulation of gingipain-derived adhesin proteins within mutant biofilms. Overall, these findings support the model that PPAD modulates *P. gingivalis* biofilm formation in a hyperfimbriated strain via citrullination of secreted proteins known to play a role in attachment and biofilm development.

EM of Δ8820 colony biofilms revealed the presence of a profuse extracellular substance. We hypothesized that the extracellular substance was a component of the biofilm matrix of Δ8820 biofilms. EM analysis confirmed that gingipain-derived adhesin proteins localized to the cells and OMVs, as well as to the matrix-like substance. Therefore, the results suggest that these secreted protein aggregates are indeed a major component of the extracellular matrix. PPAD is secreted through the T9SS and can citrullinate other proteins secreted by the T9SS. Since PPAD normally citrullinates peptides or proteins including those that are secreted by the T9SS, it is likely that the observed extracellular fibers produced by Δ8820 are made up of proteins secreted by the T9SS, specifically proteins that normally rely on citrullination for normal folding, secretion, and/or localization to the cell-surface or to OMVs. Overall, further research is needed to identify the composition of the matrix produced by the PPAD null mutant.

Although fimbriae contain many arginine residues, mass spectrometry did not predict that fimbriae were citrullinated in strain 381 under the conditions tested; though, as noted above, identification of citrullinated proteins by mass spectrometry is technically challenging, so it is not yet clear if these surface structures are modified by PPAD. Stobernack et al. showed that a clinical isolate of *P. gingivalis* tentatively citrullinated FimA and confidently citrullinated Mfa1, but, similar to our findings, neither FimA nor Mfa1 were predicted to be citrullinated in lab strain ATCC 33277.^7^ However, fimbriae can bind to arginine residues within proteins and peptides, including peptides that can be citrullinated by PPAD.^20,43^ Therefore, it is possible that even if PPAD does not citrullinate fimbriae directly in strain 381, the retention of positive arginine residues within fimbriae-associated proteins and/or peptides in the absence of PPAD may allow for a greater number of interactions with fimbriae that may in turn affect surface attachment, cell–cell interactions, and/or autoaggregation.

On the other hand, gingpains, primarily arginine gingipains (RgpA and RgpB), have long been associated with PPAD. Rgp deletion mutants have significantly fewer citrullinated proteins than their parent strains.^5^ This along with evidence showing that PPAD preferentially acts on C-terminal arginine residues supports the model that Rgp cleaves proteins at arginine residues thus freeing a C-terminal arginine residue for PPAD to citrullinate. That said, RgpA can also be citrullinated by PPAD.^7^ Our study supports previous findings that PPAD citrullinates RgpA and Kgp. Interestingly, our study and that of Stobernack et al. show that Rgp and Kgp are predicted to be citrullinated within adhesin domains, not the catalytic domain.^7^ Although citrullination of the catalytic domain of these gingipains was not detected in our study, Rgp and Kgp enzymatic activity was altered in the PPAD deletion mutant; Δ8820 produced less enzymatic activity than 381 by secreted Rgp in planktonic culture, while Δ8820 biofilms retained more Rgp and Kgp enzymatic activity than 381. It is known that the mature proteases can form complexes by non-covalently binding processed adhesin proteins.^44^ Therefore, the increase in gingipain activity in Δ8820 biofilm lysates may be due to the mature proteases binding to the positively charged adhesin proteins. Furthermore, we observed fewer adhesin proteins in Δ8820 OMV samples which, based on the dry weights of OMV preparations (unpublished data), may be due to fewer OMVs being released from the surface of the mutant cells. As a result of these findings, we posit that PPAD activity may affect the loading and/or release of OMVs and that in the absence of PPAD, OMVs and the proteins within those OMVs, including proteases and adhesin proteins, remain associated with the cell surface, promoting attachment and biofilm formation.

Expression of PPAD in strain W50 has been shown to be important for inducing alveolar bone loss in BALB/c mice, as well as in attachment and invasion of gingival fibroblasts by strain 33277;^14,26^ yet, our results show that deleting the gene that encodes PPAD in hyperfimbriated strain 381 enhances biofilm formation in monoculture. Hence, it is possible that by simply neutralizing certain charged arginine residues PPAD plays a central role in modulating colonization and pathogenesis of *P. gingivalis*, though the impact may vary depending on strain. Additionally, *P. gingivalis* is a member of the subgingival biofilm, which is comprised of several other bacterial species with which *P. gingivalis* can bind and interact. Recent publications have examined the role of PPAD in biofilm formation and interspecies, as well as interkingdom, interactions. One study, using ATCC 33277, which is a close relative of strain 381 based on genome clustering analysis, determined that deletion of PPAD did not alter biofilm formation in a single species biofilm or in a multispecies system.^45–47^ In contrast, another study using the encapsulated strain W83, found that deletion of PPAD reduced binding of W83 to *Candida albicans*.^48^ In essence different *P. gingivalis* strains appear to exhibit distinct colonization and/or biofilm phenotypes when PPAD is deleted; however, it is important to note that the growth medium used was different in each of these studies. In addition, although the chromosomal gene order between ATCC 33277 and 381 is nearly identical, strain 381 is hyperfimbriated, resulting in a robust biofilm formation phenotype. In summary, it is clear that PPAD can impact colonization in different strains, yet additional studies with a variety of isolates under the same growth conditions is required to unravel the true impact of PPAD on the basic physiology of *P. gingivalis*.

In conclusion, in this study we determined that the unique prokaryotic PPAD has a significant impact on *P. gingivalis* strain 381 biofilm formation, a key feature of the bacterium’s ability to persist and cause disease. It has been well established that *P. gingivalis* can citrullinate endogenous proteins and the results of our study demonstrate that citrullination of gingipain-derived adhesin domains limits their accumulation in and the formation of *P. gingivalis* biofilms. Further investigations into the role PPAD plays in *P. gingivalis* physiology will likely increase our understanding of the pathogenic potential of this pathobiont.

## Methods

### Bacterial strains and culture conditions

Bacterial strains and plasmids used in this study are shown in Supplementary Table 1. *P. gingivalis* strain 381 (kindly provided by Dr. Howard Kuramitsu, State University of Buffalo, Buffalo, NY) and derivatives were grown on Trypticase Soy Agar plates supplemented with 5 µg ml^−1^ hemin, 1 µg ml^−1^ menadione, and 5% defibrinated sheep blood (BAPHK) (Northeast Laboratory Services) at 37 °C in an anaerobic chamber (Coy Lab Products) with an atmosphere containing 5% hydrogen, 10% carbon dioxide, and 85% nitrogen. Planktonic cultures of *P. gingivalis* were grown in Todd Hewitt Broth (Becton, Dickinson and Company) supplemented with 5 µg ml^−1^ hemin and 1 µg ml^−1^ menadione (THBHK). *P. gingivalis* deletion mutants were maintained by supplementing media with 10 µg ml^−1^ erythromycin. *P. gingivalis* strains harboring pT-COW plasmids were maintained by supplementing media with 1 µg ml^−1^ tetracycline. For *P. gingivalis* strains grown as colony biofilms, *P. gingivalis* was grown anaerobically in THBHK for 24 h at 37 °C, sub-cultured into pre-reduced THBHK, and grown overnight. Cultures were diluted to an OD_600_ of 1.0, diluted 1:10, and then aliquots (10 µl) were spotted onto BAPHK. Plates were grown anaerobically for 4–6 days. *Escherichia coli* strains were grown in Luria Broth (LB) (Thermo Fisher Scientific) or on LB agar plates at 37 °C. *E. coli* plasmid strains were maintained by supplementing the media with 100 µg ml^−1^ ampicillin.

### Construction and complementation of mutant strain

Deletion and replacement of the entire coding region of the gene encoding PPAD (PGF_00008820) with *ermF* was achieved by generating an allelic replacement cassette with the NEBuilder HiFi DNA assembly cloning kit (New England BioLabs) using the instructions provided by the manufacturer, as previously described.^49^ All primers used in this study are presented in Supplementary Table 2. The PPAD deletion mutant (Δ8820) was complemented *in trans* by inserting a functional copy of PGF_00008820 into plasmid pT-COW under the control of the low level, constitutive *P. gingivalis groES* promoter generating plasmid pT-C8820.^50,51^ Plasmid pT-C8820 was transformed into Δ8820 by electroporation. For control strains, plasmid pT-COW was transformed into both the parent strain 381 and Δ8820 mutant by electroporation.

### Growth and imaging of biofilms

Biofilm assays in uncoated 96-well polystyrene plates were performed in a chemically defined media supplemented with 1% tryptone (CDM-T) as previously described.^28^ Fluorescent imaging was performed in the anaerobic chamber on biofilms grown on uncoated 12-well glass-bottom plates using the Invitrogen Live/Dead BacLight Bacterial Viability Kit (Thermo Thermo Fisher Scientific) and SYPRO Ruby Biofilm Matrix Stain (Thermo Thermo Fisher Scientific) as per the manufacturer’s instructions. In brief, culture supernatants (1 ml) were removed; wells were washed twice with distilled water; 1 ml of Live/Dead stain (components A [SYTO 9] and B [propidium iodide] were mixed in equal volumes and 3 µl were added per 1 ml of distilled water), SYTO 9, or SYPRO Ruby Biofilm Matrix Stain was added to each well; and then the plate was incubated in the dark for 15 min. The dye mixture was removed and water added to cover the base of the well. Images of biofilms stained with SYTO 9 and propidium iodided were acquired using a Nikon Eclipse Ti inverted fluorescence microscope setup inside the anaerobic chamber. SYTO 9 fluorescence was detected using the FITC bandpass filter cube. Propidium iodided fluorescence was detected using the Texas Red bandpass filter cube. Images of biofilms stained with SYTO 9 and SYPRO Ruby were acquired by confocal scanning laser microscopy (CLSM) using a spinning disk confocal system connected to a Leica DMIRB inverted fluorescence microscope equipped with a Photometrics cascade-cooled electron-multiplying CCD (EMCCD) camera. SYTO 9 fluorescence was detected by excitation at 488 nm and emission was collected using a 525-nm bandpass filter. The detection of SYPRO Ruby was performed using a 642-nm excitation laser and a 695-nm bandpass filter. Images were acquired using the ×63 objective. Stacked maximum intensity projection images were analyzed using Comstat2 version 2.1.^52^

### Transmission electron microscopy (TEM)

Forty microliters of 1x PBS was placed on a single *P. gingivalis* colony biofilm and suspended. Poly-l-lysine treated, glow discharged, carbon-coated Formvar 400 mesh copper grid was floated onto the suspended colony for 5 min, followed by a quick water wash. Without letting the grid dry, the grid was floated onto a 10 µl droplet of 4% paraformaldehyde for 5 min. Excess solution was dabbed off with filter paper and grid was floated on 0.5% aqueous uranyl acetate for 30 s. Stain was removed with filter paper, air dried and examined with FEI Tecnai G2 Spirit Twin TEM (FEI Corp.) and digital images were acquired with Gatan UltraScan 2*k* × 2*k* camera and Digital Micrograph software (Gatan Inc.).

### Cryo scanning electron microscopy

Cryo-SEM experiments were performed using a Quorum PP3010T cryo-transfer system (Quorum Technologies, Electron Microscopy Sciences) attached to a Hitachi SU5000FE VP-SEM (Hitachi High Technologies, America). Samples were prepared for Cryo-SEM by removal of the colony biofilm from blood agar substrate with a #11 scalpel and mounted onto a specimen shuttle containing a carbon adhesive tab (Electron Microscopy Sciences) on an aluminum stub. The sample containing shuttle was attached to the PrepDek® workstation transfer rod device, plunge frozen in slushed liquid nitrogen at −210 °C under vacuum and quickly transferred to the Cryo-preparation chamber. To remove any condensed ice from the surface gained during transfer, the sample temperature was raised to −95 °C and sublimed for 10 min. To avoid charging artifacts and to render the sample conductive, a thin layer of platinum was sputter coated for 60 s at 10 mA current in an argon atmosphere at −95 °C. The Cryo-prep chamber returned to −195 °C, at a vacuum of >10^−5^ mbar and transferred to the nitrogen gas-cooled cold stage inside the SEM chamber. The sample remained frozen during the imaging at −195 °C, under high vacuum conditions using 5–6 keV, current emission 176,000 nA, and working distance between 5 and 10 mm.

### Mass spectrometry analysis

*P. gingivalis* stationary-phase cultures were centrifuged, supernatant was removed, and cells were lysed in xTractor Buffer containing DNase I, lysozyme, and protease inhibitor (Takara Bio USA Inc.). Lysates were cleared by centrifugation at 12,000×*g* for 20 min at 4 °C and protein concentration of cleared lysates was determined using the Pierce^TM^ BCA Protein Assay Kit (Thermo Fisher Scientific). Thirty micrograms of total protein was run on a 12% SDS–PAGE gel until the dye front was 5–10 mm down the resolving gel. The gel was stained with Bio-Safe Coomassie G-250 (BioRad) and the entirety of stained protein was carefully cut from the gel and then cut into 1–2 mm cubes.

For mass spectrometry analyses, the protein samples were solubilized and digested with trypsin. The digested peptides were desalted using micro ZipTip mini-reverse phase (Millipore). Peptides derived from the protein samples were resuspended in 0.1% formic acid for mass spectrometric analysis. The mass spectrometry data acquisition was performed on an EASY-nLC 1200 ultraperformance liquid chromatography system (Thermo Fisher Scientific) connected to an Orbitrap Q-Exactive Plus instrument equipped with a nano-electrospray source (Thermo Fisher Scientific). The peptide samples were loaded to a C18 trapping column (75 μm i.d. × 2 cm, Acclaim PepMap® 100 particles with 3 μm size and 100 Å pores) and then eluted using a C18 analytical column (75 μm i.d. × 25 cm, 2 μm particles with 100 Å pore size). The flow rate was set at 250 nL/min with solvent A (0.1% formic acid in water) and solvent B (0.1% formic acid and 99.9% acetonitrile) as the mobile phases. Separation was conducted using a gradient and the full MS1 scan (*m*/*z* 350–1800) was performed on the Orbitrap with a resolution of 70,000. The MS/MS was carried out in the Orbitrap, with a quadrupole isolation window of 1.3 Da. Fragmentation of the top 10 selected peptides by high-energy collision dissociation (HCD) was done at 27% of normalized collision energy. The MS2 spectra were acquired at a resolution of 17,500 and detected through Fourier transformation of image current with the AGC target as 5e5 and the maximum injection time as 50 ms.

The tandem mass spectra were extracted from the Xcalibur.raw files and converted into mgf files using Proteome Discoverer 2.1 (Thermo Thermo Fisher Scientific). Charge state deconvolution and deisotoping were not performed. All MS/MS samples were analyzed using Mascot (Matrix Science, version 2.4.1). Mascot was set up to search the NCBInr_20130403 database (selected for Bacteria, unknown version, 14961948 entries) assuming the digestion enzyme trypsin. Mascot was searched with a fragment ion mass tolerance of 0.0100 Da and a parent ion tolerance of 10.0 ppm. Carbamidomethyl of cysteine was specified in Mascot as a fixed modification. Gln → pyro-Glu of the n-terminus, deamidated asparagine and glutamine, oxidation of methionine, and deimination of arginine were specified in Mascot as variable modifications. Scaffold (Proteome Software Inc., version 4.2.1) was used to validate MS/MS-based peptide and protein identifications. Peptide identifications were accepted if they could be established at >80% probability by the Peptide Prophet algorithm with Scaffold delta-mass correction.^53^ Protein identifications were accepted if they could be established at >90% probability and contained at least one identified peptide. Protein probabilities were assigned by the Protein Prophet algorithm.^54^ Proteins that contained similar peptides and could not be differentiated based on MS/MS analysis alone were grouped to satisfy the principles of parsimony. Proteins sharing significant peptide evidence were grouped into clusters.

### Protein expression and purification

The coding regions of RgpA adhesin domain Rgp27 and Kgp adhesin domain Kgp39 from *P. gingivalis* strain 381 were PCR amplified and cloned into pET-22b using the NEBuilder HiFi DNA assembly cloning kit (New England BioLabs) using the instructions provided by the manufacturer. Primers (Supplementary Table 2) were designed to incorporate sufficient overlapping regions at the ends of the products to permit the assembly of the Rgp27 or Kgp39 coding regions (inserts) with the PCR-linearized vector pET-22b. Products were generated using Phusion High-Fidelity PCR Master Mix with HF Buffer (New England BioLabs). The inserts were mixed 2:1 with the vector backbone and incubated with the NEB Assembly Master Mix according to the manufacturer’s instructions. The assembled product was used to transform NEB 5α chemically competent *E. coli* (New England BioLabs) thereby generating pET22b-Rgp27 and pET22b-Kgp39. The inserts were sequenced using T7 forward and reverse primers. Verified plasmids were then transformed into *E. coli* strain BL21(DE3) (New England BioLabs).

*E. coli* strain BL21(DE3) containing pET22b-Rgp27 or pET22b-Kgp39 was grown in LB broth containing 100 µg ml^−1^ ampicillin at 37 °C on a platform shaker at 250 rpm until the cultures reached an OD_600_ of 1.0. Cultures were then induced with IPTG at a final concentration of 1 mM and grown for an additional 4 h. Cells were then pelleted at 4700 rpm for 5 min. and the pellets were frozen at −20 °C. To purify proteins, 8 ml guanidinium lysis buffer (6 M guanidine hydrochloride, 500 mM NaCl, 20 mM sodium phosphate pH 7.8) per 50 ml culture pellet was added and cells were lysed by rocking for 10 min at room temperature. Cell lysate was sonicated on ice with three 5 s pulses at high intensity. Lysate was centrifuged at 3000×*g* for 15 min at 4 °C. Suspended HisPur Ni-NTA-agarose (Thermo Thermo Fisher Scientific) was added to a Pierce® centrifuge column (Thermo Thermo Fisher Scientific). Once the resin settled, the supernatant was gently removed and the resin was washed once in sterile dH_2_O then equilibrated twice in denaturing binding buffer (8 M urea, 500 mM NaCl, 20 mM sodium phosphate pH 7.8). Lysate was transferred to the prepared purification column and incubated for 30 min at room temperature with rocking. The column was washed two times with denaturing binding buffer for 2 min. The column was then washed two times with denaturing wash buffer (8 M urea, 500 mM NaCl, 20 mM sodium phosphate pH 6.0) for 2 min followed by washing four times with native wash buffer (20 mM imidazole, 0.5 M NaCl, 50 mM NaH_2_PO_4_ pH 8.0) for 2 min. Ni-bound protein was eluted with native elution buffer (250 mM imidazole, 0.5 M NaCl, 50 mM NaH_2_PO_4_ pH 8.0). Proteins were dialyzed overnight in 0.1 M Tris/HCl (pH 7.5) containing 10% glycerol. Proteins were stored at −20 °C.

### PPAD enzymatic activity

Stationary-phase cultures were diluted to the same OD_600_ and the PPAD enzymatic activity assay was setup in a 96-well PCR plate (Bio-Rad Laboratories, Inc.) and measured as previously described.^55^ In brief, 10 µl of culture was added to 35 µl incubation buffer (0.1 M Tris–HCl buffer [pH 7.5] and 5 mM DTT) for experimental wells or 40 µl incubation buffer for control wells. 5 µl of substrate (BAEE or l-arginine) was added to each experimental well to a final concentration of 5 mM. 5 µl of substrate was added to 45 µl incubation buffer for substrate only controls. The plate was incubated at 37 °C in a thermocycler for 30 min. 150 µl of freshly prepared citrulline detection reagent (1 volume of solution A [80 mM 2,3-butanedione monoxime and 2 mM thiosemicarbazide] and 3 volumes of solution B [3 M H_3_PO_4_, 6 M H_2_SO_4_, and 2 mM NH_4_Fe(SO_4_)_2_·12 H_2_O]) was added to each well, then the plate was incubated at 95 °C in a thermocycler for 15 min. The samples were then transferred to a 96-well flat-bottom plate (Corning, Inc.) and enzymatically produced citrulline was detected at an absorbance of 540 nm.

To test the ability of *P. gingivalis* to citrullinate rRgp27 and rKgp39, the following modifications were made to the above protocol. Stationary cultures were centrifuged and pellets were re-suspended to an OD_600_ of 1.0 in incubation buffer. rRgp27, rKgp39, and BAEE (positive control) were diluted to 0.15 mg ml^−1^ in incubation buffer. 11 µl of *P. gingivalis* was added to 11 µl of incubation buffer (negative control), rRgp27, rKgp39, or BAEE (positive control) per well of a 96-well PCR plate. 11 µl rRgp27, rKgp39, or BAEE was added to 11 µl incubation buffer as substrate only controls. The plate was sealed and incubated at 37 °C in a thermocycler for 1 h. After incubation, the seal was removed and 66 µl of freshly prepared citrulline detection reagent was added to each well.

### Immunoblots

For planktonic samples, *P. gingivalis* was grown in THBHK for 24 h, sub-cultured into pre-reduced THBHK, and grown to early exponential, mid-exponential, late exponential, and stationary phases. Cells were centrifuged, supernatant was removed, and cells were resuspended to a final OD_600_ of 1.0 in dH_2_O. For biofilm samples, 24 h biofilms were washed twice in dH_2_O and then lysed with scraping in xTractor Buffer containing DNase I, lysozyme, and protease inhibitor (Takara Bio USA Inc.). Lysates were cleared by centrifugation at 12,000×*g* for 20 min at 4 °C and protein concentration of cleared lysates was determined using the Pierce^TM^ BCA Protein Assay Kit (Thermo Fisher Scientific). Planktonic or biofilm samples were mixed with 2X SDS sample buffer and denatured by heating at 100 °C for 5 min. Planktonic samples (10 µl) or biofilm samples (5 µg) were then electrophoresed on a 12% polyacrylamide SDS–PAGE gel. For western blot analysis, proteins were transferred to a nitrocellulose membrane. The membrane was washed once for 5 min in TBS containing 0.1% Tween 20 (TBS-T) then blocked for 1 h in 5% milk in TBS-T with rocking at room temperature. Primary anti-FimA (kindly provided by Dr. Ashu Sharma, University of Buffalo, Buffalo, New York), anti-Mfa1 (kindly provided by Dr. Richard Lamont, University of Louisville, Louisville, Kentucky), or anti-adhesin (kindly provided by Dr. Mike Curtis, King’s College London, London, UK) antiserum was added 1:5000, 1:10,000, or 1:500, respectively, and incubated with the membrane for 1 h with rocking at room temperature. After washing the membrane in TBS-T for 5 min three times, the membrane was incubated with peroxidase-conjugated anti-rabbit IgG antibody at a dilution of 1:2000 for 1 h with rocking at room temperature. The membrane was washed three times in TBS-T for 5 min before detecting bands using SuperSignal^R^ West Pico Chemiluminescent Substrate (Thermo Fisher Scientific). Bands were quantified by measuring the band intensity in Image Lab version 5.2.1.

Previous experiments showed that the anti-FimA and anti-Mfa1 antibodies are specific and hybridized to proteins at 40 and 75 kDa, respectively. Occasionally it was observed that with increasing exposure time anti-Mfa1 hybridized to proteins below 75 kDa and above 50 kDa (Supplementary Figure 3). Since these antibodies were well established to be specific, a single membrane was cut between the 75 kDa and the 50 kDa marker bands. The upper portion of the membrane was probed with the anti-Mfa1 antibody while the lower portion was probed with the anti-FimA antibody. For experiments using the anti-adhesin antibody, it is known that this antibody binds to multiple bands corresponding to the various gingipain-derived adhesin proteins. The anti-adhesin antibody did not bind to proteins above 50 kDa under the conditions tested. Therefore, a single membrane was cut between the 75 kDa and the 50 kDa marker bands. The upper portion of the membrane was probed with the anti-Mfa1 antibody as a loading control while the lower portion was probed with the anti-adhesin antibody. All bands identified by probing with the anti-adhesin antibody are shown.

### Scanning electron microscopy (SEM)

Cells from colony biofilms were collected from blood agar plates with 4% paraformaldehyde in 1X PBS, pH 7.24. Fixed cells were processed with the aid of a Pelco BioWave Pro laboratory microwave. The cells were deposited onto poly-l-lysine treated 13 mm Thermanox plastic coverslips, rinsed in PBS, and incubated for 20 min on blocking solution (1% non-fat dry milk, 0.5% cold water fish skin gelatin, 0.01% Tween-20 in PBS [pH 7.2]). After a PBS wash, the Thermanox coverslip containing cells was incubated for 1 h at room temperature with primary antibody at a dilution of 1:200, washed with PBS three times, and incubated for 1 h with 18 nm colloidal gold-conjugated goat-anti-rabbit IgG diluted 1:20 (Jackson ImmunoResearch). Subsequently, washed with PBS, fixed with 2% glutaraldehyde, washed with water, and dehydrated in a graded ethanol series (25%, 50%, 75%, 95%, 100%) and critical point dried. Thermanox coverslip were mounted onto carbon adhesive tabs on aluminum specimen mount and carbon coated (Cressington 328/308R). Secondary electron (SE) and backscatter electron (BSE) digital micrographs were acquired using SEM (SU5000 VP-SEM, Hitachi High-Technologies, America).

### Gingipain assay

The activity of arginine and lysine gingipains was assessed as previously described.^56^ Briefly, *P. gingivalis* was grown anaerobically in THBHK for 24 h. Cultures were either diluted into pre-reduced THBHK and grown to mid-exponential phase or diluted into CDM-T and grown in a polystyrene flat-bottom plate for 24 h. Planktonic cultures were then normalized to an OD_600_ of 1.0 and 1 ml of each culture centrifuged to pellet the cells. The supernatants were transferred to a separate tube while the pellets were resuspended in assay buffer (200 mM Tris, 5 mM CaCl_2_, 150 mM NaCl, and 10 mM l-cysteine at pH 7.6). 24 h biofilms were washed twice in dH_2_O and then lysed with scraping in xTractor Buffer containing DNase I, lysozyme, and protease inhibitor (Takara Bio USA Inc.). Lysates were cleared by centrifugation at 12,000×*g* for 20 min at 4 °C and protein concentration of cleared lysates was determined using the Pierce^TM^ BCA Protein Assay Kit (Thermo Fisher Scientific). Biofilm lysates were normalized to a protein concentration of 5 µg µl^−1^. Cells, supernatants, or biofilm lysates were diluted 1:10 in assay buffer and then serially diluted in assay buffer across a 96-well microtiter plate. The initial absorbance at 405 nm was measured and the plates were then incubated at 37 °C for 10 min to equilibrate the temperature. N-α-benzoyl-L-arginine-p-nitroanilide (BAPNA) or N-α-acetyl-L-lysine-p-nitroanilide (ALPNA) was added to the wells at a final concentration of 1 mM and the microtiter plates were incubated for 2 h at 37 °C. The final absorbance (*A*_405_) of the wells was recorded and the difference between the initial and final absorbance was determined.

### Reporting summary

Further information on experimental design is available in the Nature Research Reporting Summary linked to this article.

## Supplementary information

Supplemental Information

Reporting Summary

## Data Availability

The authors declare that the data supporting the findings of this study are available within the paper and its supplementary information files or from the corresponding author upon request.

## **Supplementary information**

**Supplementary information** accompanies the paper on the *npj Biofilms and Microbiomes* website (10.1038/s41522-019-0081-x).
