# Supplementary material for: Citrullination mediated by PPAD constrains biofilm formation in P. gingivalis strain 381
Source: NPJ Biofilms Microbiomes. 2019 Feb 7;5:7. doi: 10.1038/s41522-019-0081-x (PMC6367333; doi:10.1038/s41522-019-0081-x)
Supplement: Supplementary file 1 — Supplemental Information [file 41522_2019_81_MOESM1_ESM.pdf]

Supplementary Table 1 Strains and plasmids used in this study

**Strain or plasmid (relevant genotype or phenotype)**	**Source or reference**
P. gingivalis strains	
381 (wild type)	H. Kuramitsu, State University of Buffalo, Buffalo, NY
ΔPGF_00008820::Erm (Em^r) in strain 381	This study
Δ*rgpA*::Erm (Em^r) in strain 381	This study
Δ*kgp*::Erm (Em^r) in strain 381	This study
E. coli strains	
NEB 5α	NEB
BL21(DE3)	NEB
Plasmids	
pT-COW (Cb^r Tc^r)	45
pT-C8820 (Cb^r Tc^r)	This study
pET-22b (Am^r)	Novagen
pET22b-Hgp27 (Am^r)	This study
pET22b-Kgp39 (Am^r)	This study
pTgroES-HU (Cb^r Tc^r)	46

Supplementary Table 2 Primers used in this study

**Primer purpose and name**	**Sequence (5'-3')**
PGF_00008820 mutant construction, verification, and complementation	
1kb upstream PGF_00008820 F	GTAGGTCTCTGCGAACCTC
1kb upstream PGF_00008820 R	CTTTTTTGTCATTGTTTGATATGTTTTATGATGTTATGAATATG
PGF_00008820 KO *ermF* F	ATATCAAACAATGACAAAAAAGAAATTGCCC
PGF_00008820 KO *ermF* R	AAGGCCCCCTACGAAGGATGAAATTTTTCAG
1kb downstream PGF8820 F	CTTCGTAGGGGGCCTTATTTGAGAATAC
1kb downstream PGF8820 R	GTAAGGGTTCGCTTAAGATC
ΔPGF8820::*ermF* seq F	GTAGGTCTCTGCGAACCTC
ΔPGF8820::*ermF* seq R	GTAAGGGTTCGCTTAAGATC
pT-COW F	AATGGAAGCCGGCGGCAC
pT-COW R	TACACGGTGCCTGACTGCG
groES F	TGCTAACGCAGTCAGGCACCGTGTAATTGGATAGATGCCCTGC
groES R	GAACATATGATAATGTTGCTTGGTTTGTTATTG
PGF_00008820	AAACCAAGCAACATTATCATATGTTCAGGAACATATTC
PGF_00008820	TAGCGAGGTGCCGCCGGCTTCCATTTTATTTGAGAATTTTCATTGTCTC
pT-COW seq F	CAGTGAGGATATTGACGCTTATTTCG
pT-COW seq R	GAGTGGTGAATCCGTTAGCG
RgpA mutant construction and verification	
1kb upstream *rgpA* F	GTCTCGGATGGCATCGGG
1kb upstream *rgpA* R	TCTTTTTTGTCATTTTGATGAAATTAGAATATGATTTTACTGTGTAGTAC
rgpA KO *ermF* F	TTTCATCAAAATGACAAAAAAGAAATTGCC
rgpA KO *ermF* R	CAAGACAGAACTACGAAGGATGAAATTTTTC
1kb downstream *rgpA* F	TCCTTCGTAGTTCTGTCTTGGACTCGGAG
1kb downstream *rgpA* R	AGTACTTCACCCGTATCTATCTC
Δ*rgpA*::*ermF* seq F	CTATGTAGCGGCGGAAAAATG
Δ*rgpA*::*ermF* seq R	GTCCACTGTGTGCACCAATAC
Kgp mutant construction and verification	
1kb upstream *kgp* F	CAGCTTTCTCTCAGCCGAG
1kb upstream *kgp* R	TCTTTTTTGTCATACTTTAAAACAATTTATGGTCGTG
kgp KO *ermF* F	GTTTTAAAGTATGACAAAAAAGAAATTGCC
kgp KO *ermF* R	CAAGACAGAACTACGAAGGATGAAATTTTTC
1kb downstream *kgp* F	TCCTTCGTAGTTCTGTCTTGGACTCGGAG
1kb downstream *kgp* R	AACCCAACGGAAGCATTAC
Δ*kgp*::*ermF* seq F	TAGACCGAATTCTATCCCACAA
Δ*kgp*::*ermF* seq R	CAAACTATTGGCGCAGTTTAAC
pET22b-Hgp27 and pET22b-Kgp39 construction and verification	
pET22b F	CACCACCACCACCACCAC
pET22b R	CATATGTATATCTCCTTCTTAAAGTTAAACAAAATTATTTC
pET22b-Hgp27 F	TTTAAGAAGGAGATATACATATGAAGGTTGTGCTCGCAGCAG
pET22b-Hgp27 R	CAGTGGTGGTGGTGGTGGTGCTTTACAGCGAGTTTCTCTACGTAAG
pET22b-Kgp39 F	TTTAAGAAGGAGATATACATATGAAGAAGGCAGAAGCTTCC
pET22b-Kgp39 R	CAGTGGTGGTGGTGGTGGTGTTTTGCCGTAATCGTCTC
T7 F	GCGAAATTAATACGACTCACTAT
T7 R	CTCAAGACCCGTTTAGAGG

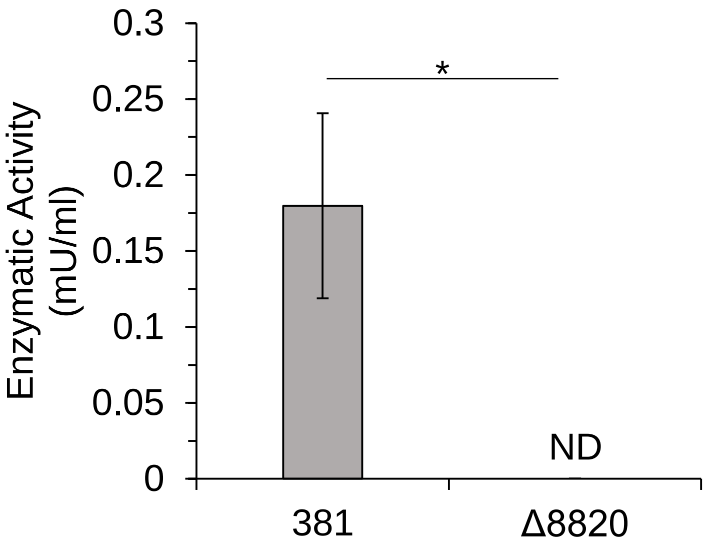

Supplementary Figure 1 Deletion of PGF_00008820 depletes peptidylarginine deiminase enzymatic activity. 381 and Δ8820 were grown to stationary phase and 10 µl aliquots were used to test enzymatic activity of PPAD. Data represent the average of three independent experiments (n=9). Error bars represent the standard deviation. The data were analyzed using the Student's two-tailed t-test. *p<0.05. ND, none detected.

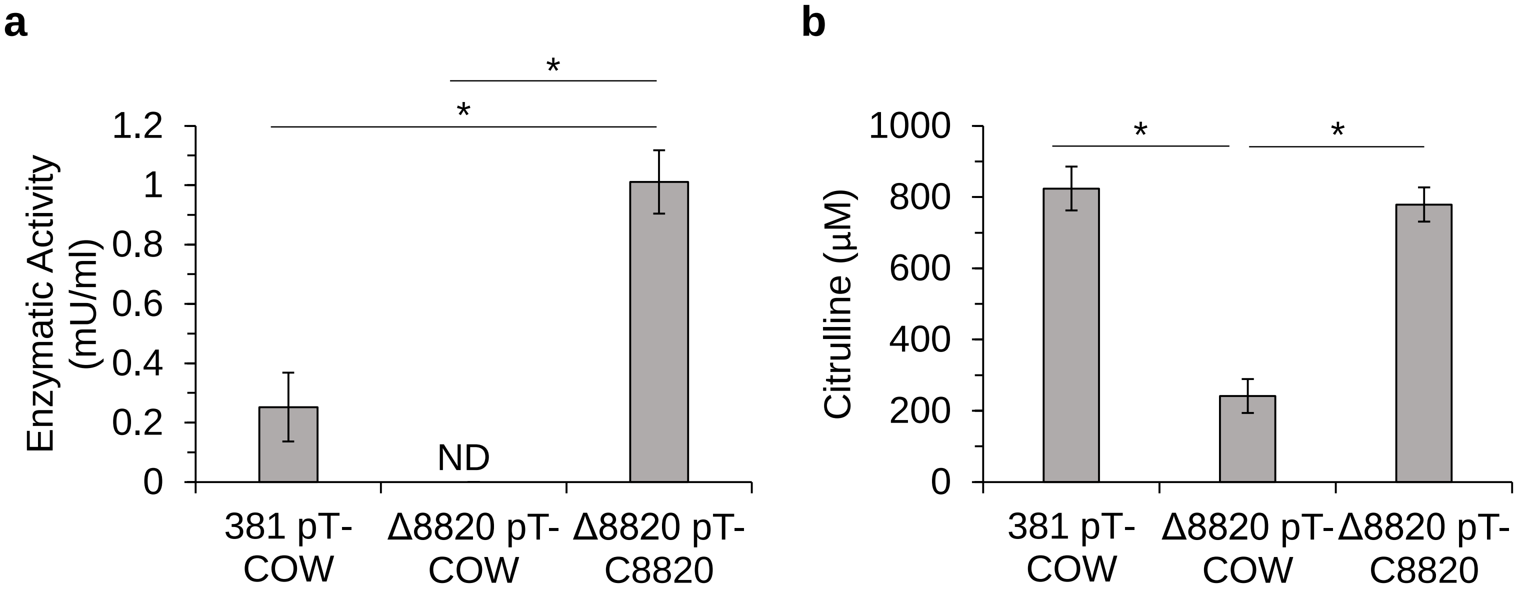

Supplementary Figure 2 Complementation restores PPAD enzymatic activity. All strains were grown to stationary phase and 10 µl aliquots were used to test (**a**) enzymatic activity of PPAD or (**b**) the concentration of citrulline in cultures. Complementation of Δ8820 restored PPAD enzymatic activity. Although the enzymatic activity of Δ8820 pT-C8820 was greater than 381 pT-COW, the concentration of citrulline in stationary cultures was the same. Data represent the average of three independent experiments (n=9). Error bars represent the standard deviation. Data represent the average of three independent experiments. Error bars represent the standard deviation. The data were analyzed by ANOVA with Bonferroni post tests. *p<0.05. ND, none detected.

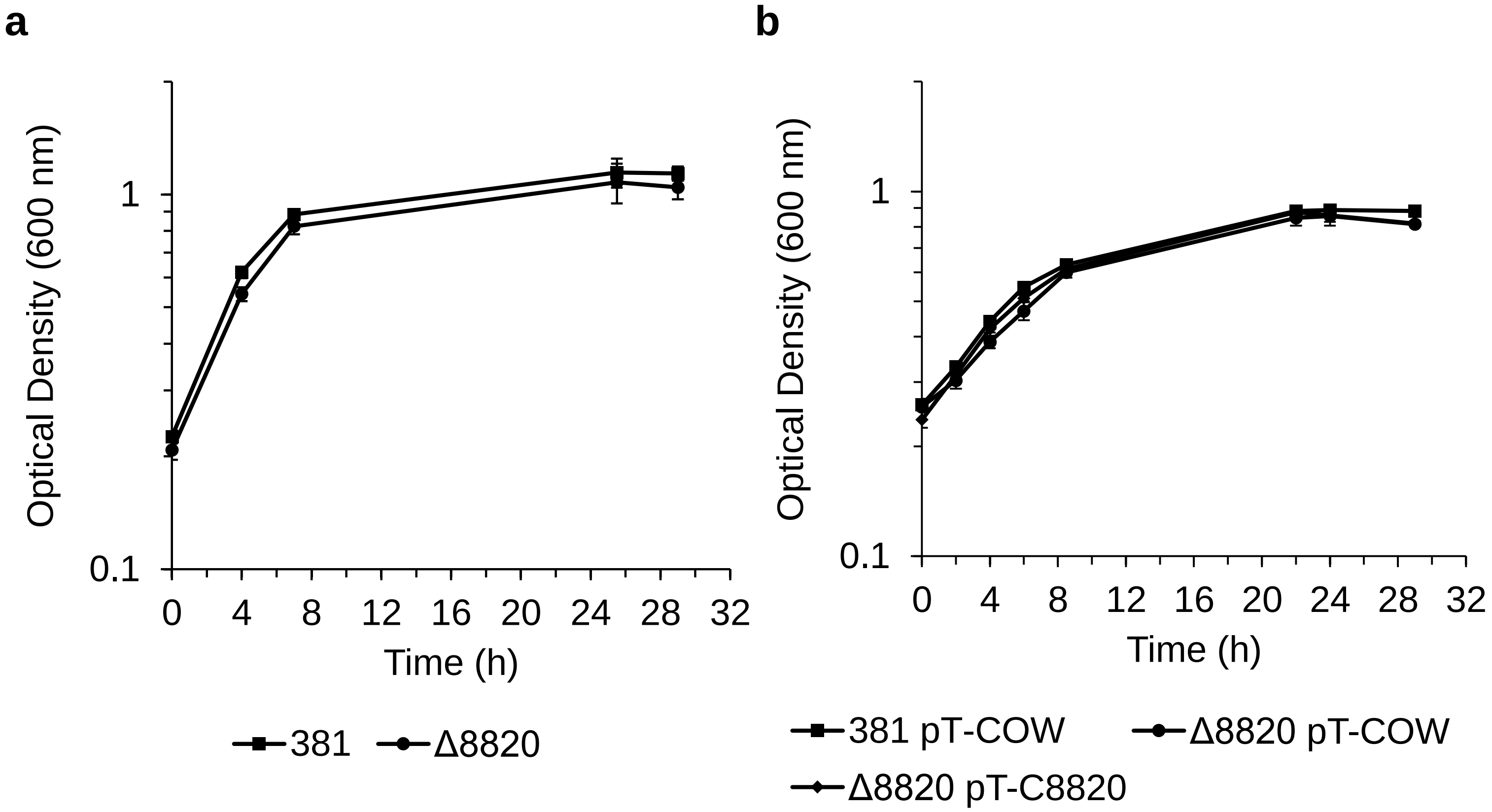

Supplementary Figure 3 (**a**) Growth curves of 381 and Δ8820 grown in chemically defined media supplemented with 1% tryptone (CDM-T). (**b**) Growth curves of 381 and Δ8820 containing pT-COW (empty vector controls) and complemented Δ8820 (pT-C8820) grown in CDM-T in the presence of tetracycline. Data represent the average of three biological replicates (n=3). Error bars represent the standard deviation.

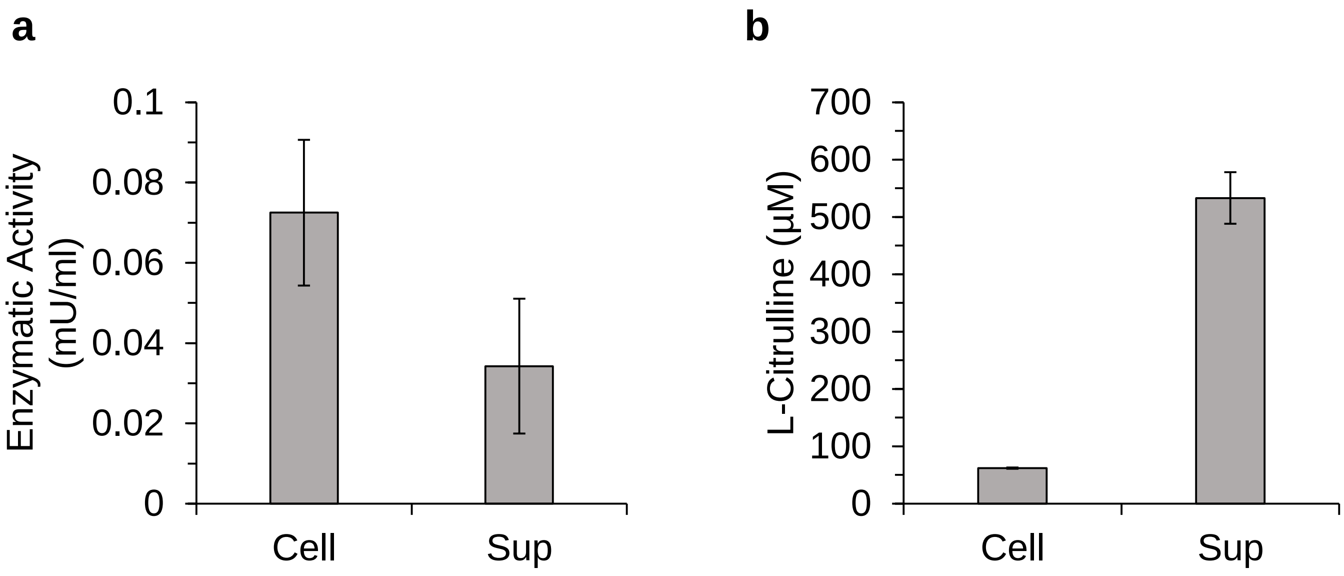

Supplementary Figure 4 The majority of PPAD enzymatic activity is cell-associated, while the majority of citrulline is found in the supernatant. (**a**) PPAD enzymatic activity and (**b**) the concentration of citrulline present on cells and in the supernatant was measured using a colorimetric assay and an L-citrulline standard curve. Data in (**a**) and (**b**) are averages of three independent experiments (n=9). Error bars represent the standard deviation.

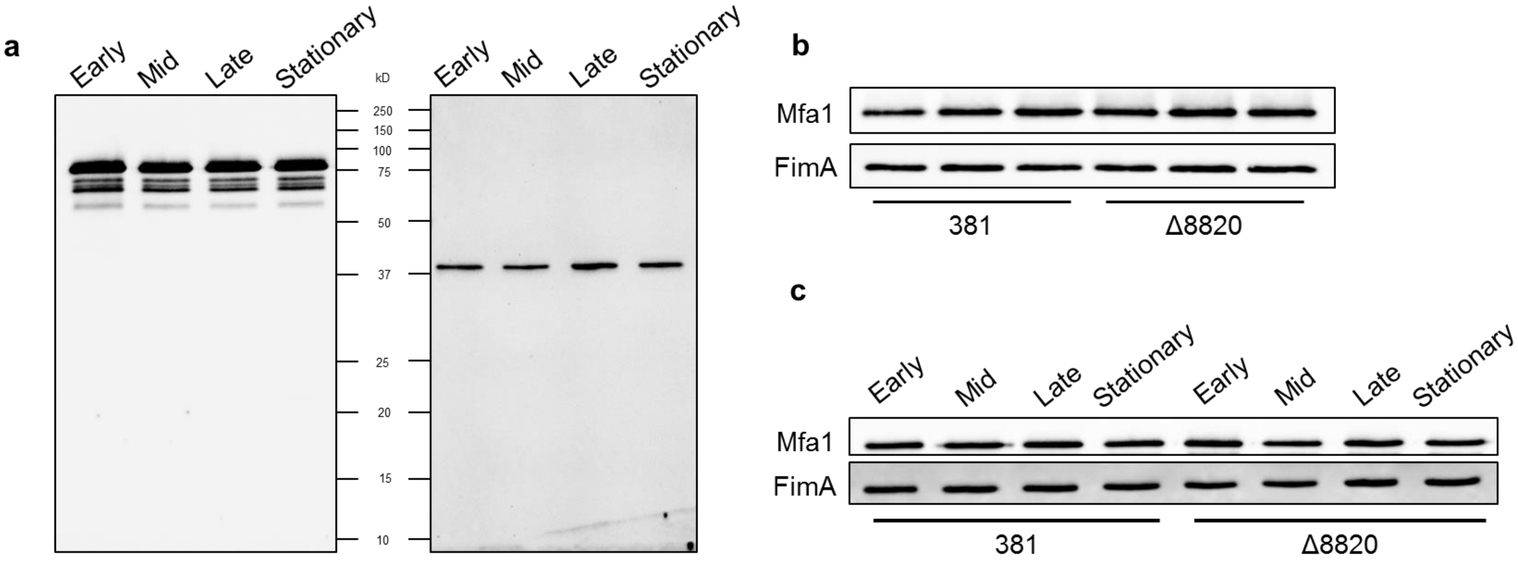

Supplementary Figure 5 Deletion of PPAD does not affect fimbriae protein expression. (**a**) Specificity of (left) anti-Mfa1 and (right) anti-FimA antibodies was tested against 381 planktonic cell samples. Anti-Mfa1 primarily hybridizes to a band at 75 kD, but occasionally binds to proteins below 75 kD and above 50 kD, especially with increasing exposure time. Anti-FimA specifically binds a protein at 40 kD. Major (FimA) and minor (Mfa1) fimbriae present in (**b**) 24 h biofilm lysates and (**c**) planktonic cell samples were analyzed by western blot. Samples derive from the same experiment and the blots were processed in parallel.

Rgp27

KVVLAADNVWGDNTGYQFLLDADHNTFGSVIPATGPLFTGTASSNLYSANFEYLIPANADPV
VTTQNIIVTGQGEVVIPGGVYDYCITNPEPASGKMWIAGDGGNQPA**R**YDDFTFEAGKKYTF
TM**RR**AGMGDGTDMEVEDDSPASYTYTVY**R**DGTKIKEGLTETTY**R**DAGMSAQSHEYCVEV
KYAAGVSPKVCVDYIPDGVADVTAQKPYTLTVVGKTITVTCQGEAMIYDMNG**RR**LAAG**R**NT
VVYTAQGGYYAVMVVVDGKSYVEKLAVK

Kgp39

KKAEAS**R**EVK**R**IGDGLFVTIEPANDV**R**ANEAKVVLAADNVWGDNTGYQFLLDADHNTFGS
VIPATGPLFTGTASSNLYSANFEYLIPANADPVVTTQNIIVTGQGEVVIPGGVYDYCITNPEPA
SGKMWIAGDGGNQPA**R**YDDFTFEAGKKYTFTM**RR**AGMGDGTDMEVEDDSPASYTYTVY
RDGTKIQEGLTATTFEEDGVAAGNHEYCVEVKYTAGVSPKVCKDVTVEGSNEFAPVQNLTG
SAVGQKVTLKWDAPNGTPNPNPNPNPGTTTLSESFENGIPASWKTIDADGDGHGWKPGN
APGIAGYNSNGCVYSESFGLGGIGVLTPDNYLITPALDLPNGGKLTFWVCAQDANYASEHY
AVYASSTGNDASNFTNALLEETITAK

Supplementary Figure 6 Gingipain-derived adhesin proteins are predicted to be citrullinated based on mass spectrometry. Shown are the sequences of Rgp27 and Kgp39 with all arginine residues indicated in bold and the arginine residues predicted to be citrullinated indicated in red and underlined.

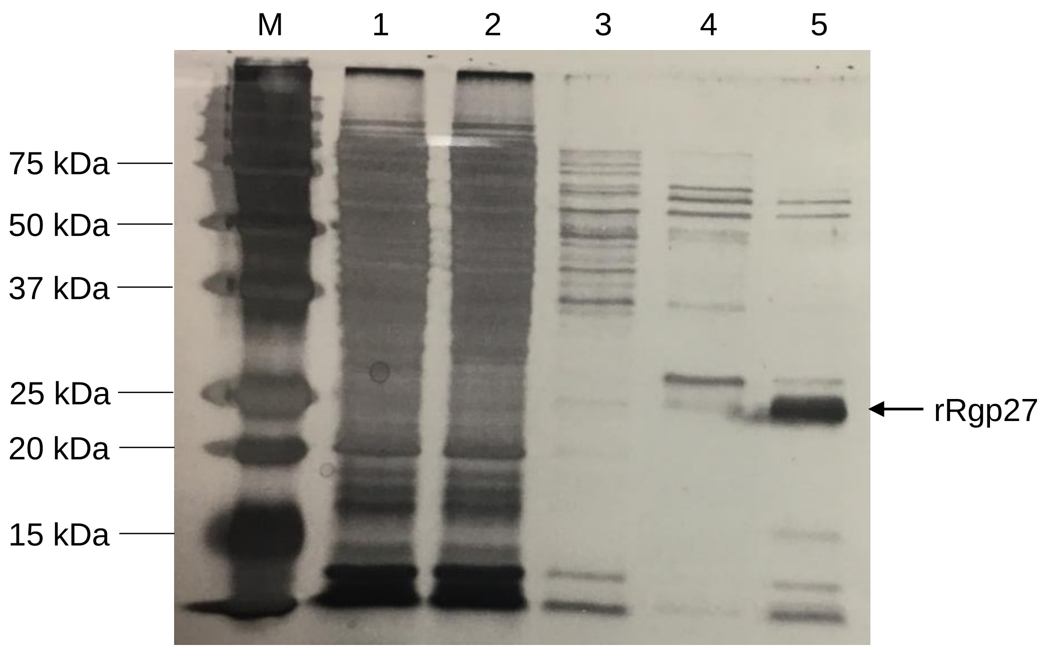

Supplementary Figure 7 Silver stain of purified recombinant adhesin domain Rgp27. Purified samples and upstream fractions were run on SDS PAGE gels and stained by silver stain to assess purity of the protein. M: Precision Plus Protein Dual Color standards, 1: BL21(DE3) pET22b-Rgp27 lysate pre Ni-NTA, 2: BL21(DE3) pET22b-Rgp27 lysate post Ni-NTA, 3: Wash, 4: Purified eluent, 5: Purified rRgp27

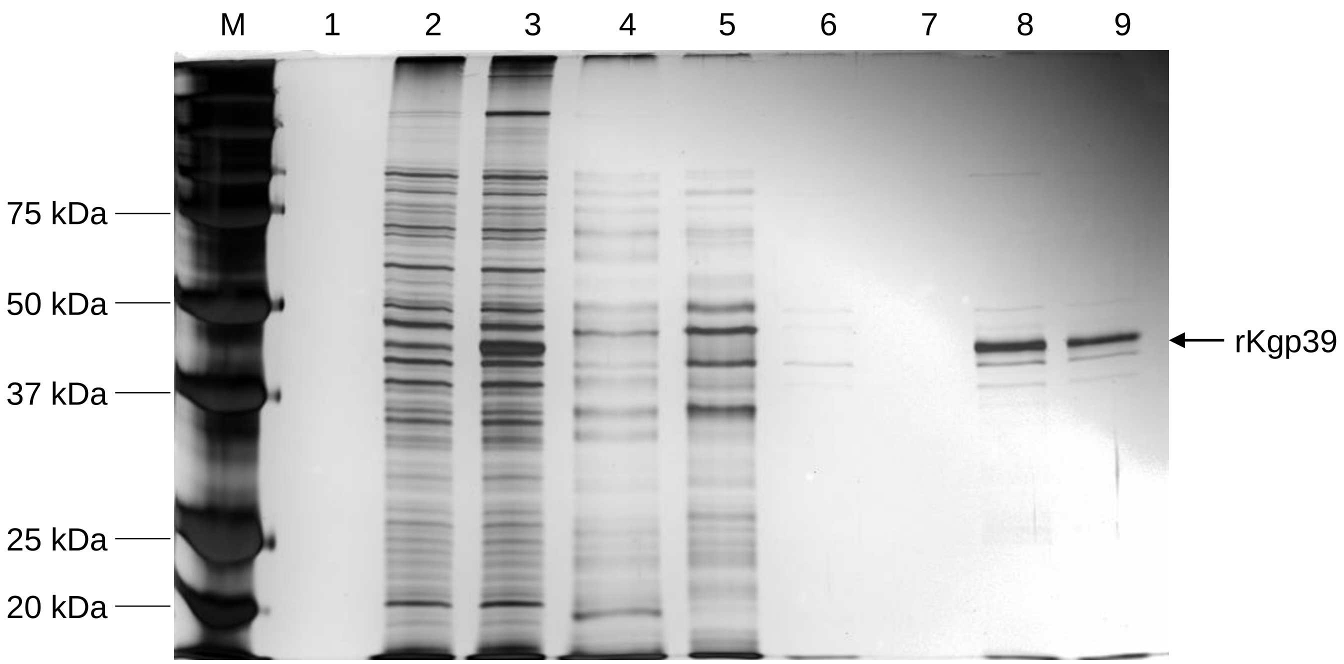

Supplementary Figure 8 Silver stain of purified recombinant adhesin domain Kgp39**.** Purified samples and upstream fractions were run on SDS PAGE gels and stained by silver stain to assess purity of the protein. M: Precision Plus Protein Dual Color standards, 1: N/A, 2: BL21(DE3) pET22b-Kgp39 pre-induction, 3: BL21(DE3) pET22b-Kgp39 post 4 h induction with 1 mM IPTG, 4: Denaturing Binding Buffer, 5: Denaturing Wash Buffer (first wash), 6: Native Wash Buffer (first wash), 7: Native Wash Buffer (fourth wash), 8: Purified rKgp39 (first elution), 9: Purified rKgp39 (second elution)

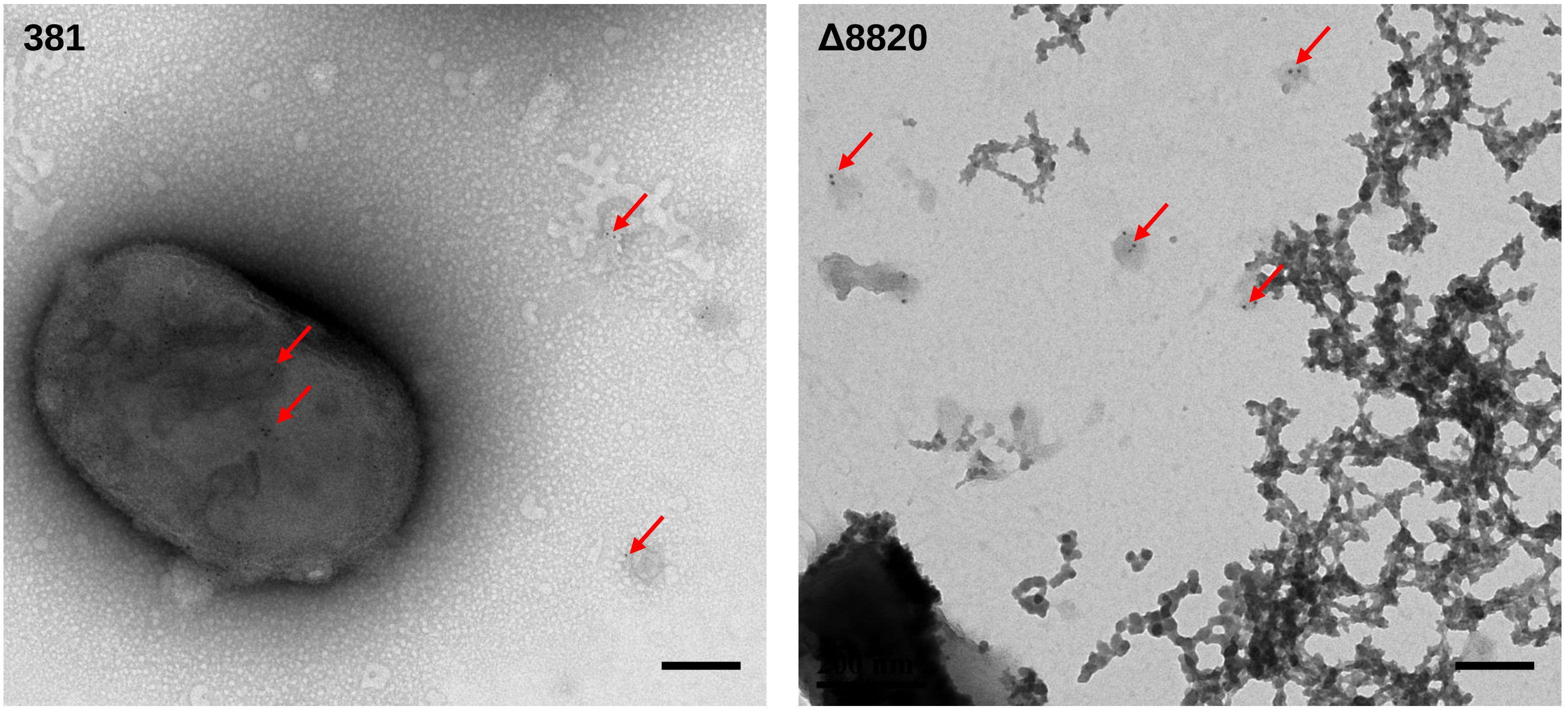

Supplementary Figure 9 Gingipain-derived adhesin proteins localize to the cell surface and OMV-like structures. Suspended 381 and Δ8820 colony biofilms grown on plates were negatively stained with 0.5% aqueous uranyl acetate, treated with the anti-adhesin primary antibody, and then images were acquired by immunogold labeling and TEM. Arrows indicate representative colloidal gold particles. Scale bar: 200 nm

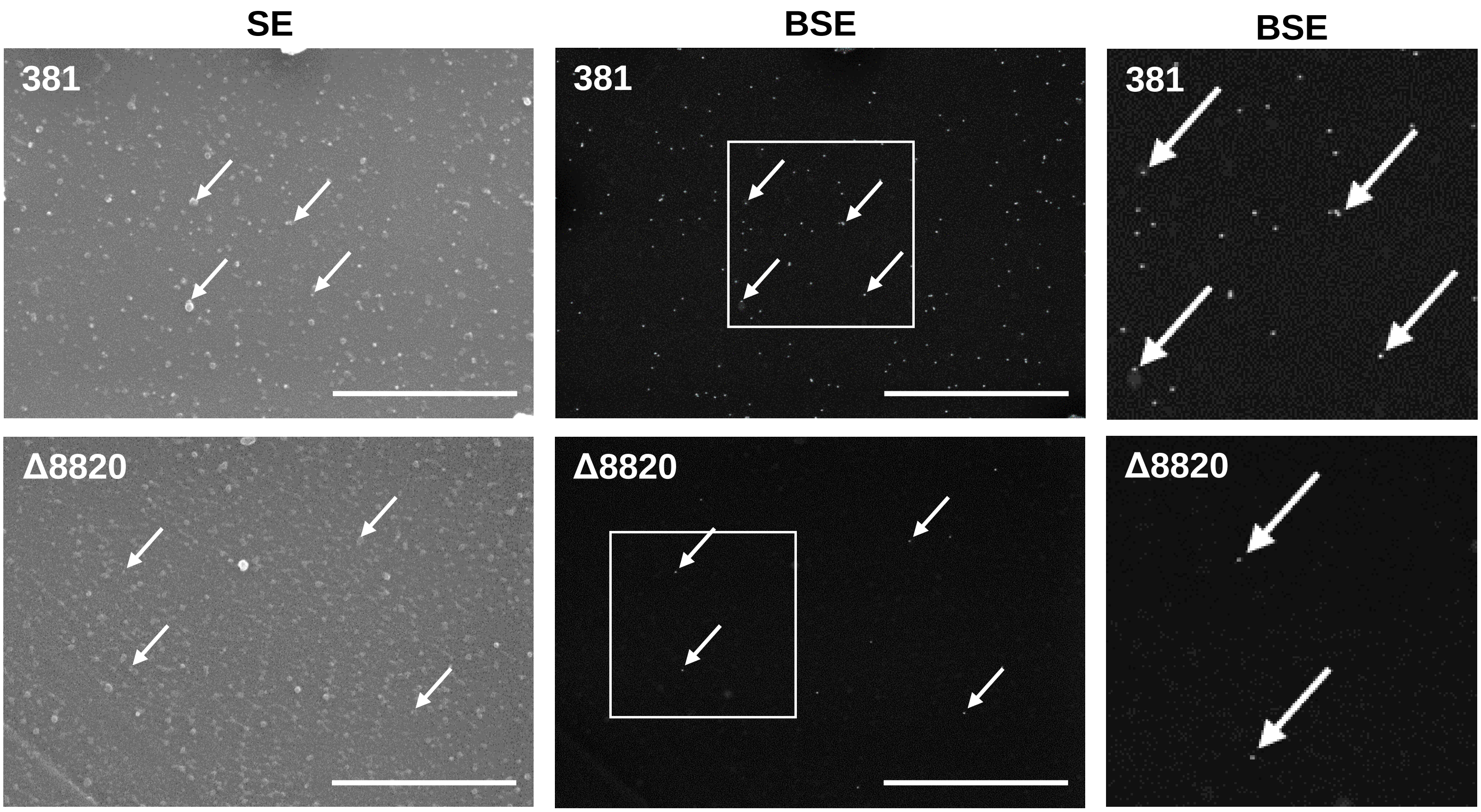

Supplementary Figure 10 OMV-associated gingipain-derived adhesin proteins were more often observed in 381 samples than Δ8820 samples. Suspended 381 and Δ8820 colony biofilms were fixed and treated with the anti-adhesin primary antibody. Images were acquired by immunogold labeling and SEM. Arrows indicate representative colloidal gold particles. SE: Secondary electron, BSE: Backscatter electron, Scale bar: 2 µm
